# Correlation of Socioeconomic and Environmental Factors With Incidence of Crohn Disease in Children and Adolescents: Systematic Review and Meta-Regression

**DOI:** 10.2196/48682

**Published:** 2024-03-25

**Authors:** Jens Weidner, Ingmar Glauche, Ulf Manuwald, Ivana Kern, Ines Reinecke, Franziska Bathelt, Makan Amin, Fan Dong, Ulrike Rothe, Joachim Kugler

**Affiliations:** 1 Institute for Medical Informatics and Biometry Medical Faculty Carl Gustav Carus TU Dresden Dresden Germany; 2 Faculty of Applied Social Sciences University of Applied Sciences (FHD) Dresden Germany; 3 Institute and Policlinic for Occupational and Social Medicine Department of Health Sciences/Public Health, Medical Faculty Carl Gustav Carus TU Dresden Dresden Germany; 4 Thiem-Research GmbH Cottbus Germany; 5 Department for Trauma Surgery and Orthopaedics Park-Klinik Weissensee Berlin Germany; 6 GWT of TUD Dresden Germany

**Keywords:** Crohn’s disease, inflammatory bowel disease, pediatric, children, adolescents, environmental factors, Crohn disease, gastroenteritis, inflammatory bowel diseases, bowel disease, digestive system, gastrointestinal disease

## Abstract

**Background:**

The worldwide incidence of Crohn disease (CD) in childhood and adolescence has an increasing trend, with significant differences between different geographic regions and individual countries. This includes an increase in the incidence of CD in countries and geographic regions where CD was not previously prevalent. In response to the increasing incidence, the pediatric care landscape is facing growing challenges.

**Objective:**

This systematic review and meta-analysis were undertaken to comprehensively delineate the incidence rates of CD in pediatric populations across different countries and to explore potential influencing factors.

**Methods:**

We performed a systematic review of PubMed and Embase (via Ovid) for studies from January 1, 1970, to December 31, 2019. In addition, a manual search was performed in relevant and previously published reviews. The results were evaluated quantitatively. For this purpose, random effects meta-analyses and meta-regressions were performed to investigate the overall incidence rate and possible factors influencing the incidence.

**Results:**

A qualitative synthesis of 74 studies was performed, with 72 studies included in the meta-analyses and 52 in the meta-regressions. The results of our meta-analysis showed significant heterogeneity between the individual studies, which cannot be explained by a sample effect alone. Our findings showed geographical differences in incidence rates, which increased with increasing distance from the equator, although no global temporal trend was apparent. The meta-regression analysis also identified geographic location, UV index, and Human Development Index as significant moderators associated with CD incidence.

**Conclusions:**

Our results suggest that pediatric CD incidence has increased in many countries since 1970 but varies widely with geographic location, which may pose challenges to the respective health care systems. We identified geographic, environmental, and socioeconomic factors that contribute to the observed heterogeneity in incidence rates. These results can serve as a basis for future research. To this end, implementations of internationally standardized and interoperable registries combined with the dissemination of health data through federated networks based on a common data model, such as the Observational Medical Outcomes Partnership, would be beneficial. This would deepen the understanding of CD and promote evidence-based approaches to preventive and interventional strategies as well as inform public health policies aimed at addressing the increasing burden of CD in children and adolescents.

**Trial Registration:**

PROSPERO International prospective register of systematic reviews CRD42020168644; https://www.crd.york.ac.uk/PROSPERO/display_record.php?RecordID=168644

**International Registered Report Identifier (IRRID):**

RR2-10.1136/bmjopen-2020-037669

## Introduction

Crohn disease (CD), ulcerative colitis (UC), and indeterminate colitis are chronic inflammations of the gastrointestinal tract and are summarized under inflammatory bowel disease (IBD). Since the beginning of the 21st century, a progression in incidence, mainly due to CD, has been observed in both industrialized and emerging countries [1,2] IBD is an immune-mediated disease that can affect people of all ages. However, about 20% of IBD cases are diagnosed before one is 20 years old, with an adverse shift in the age of diagnosis to early childhood years. Approximately 25% of children and adolescents are younger than 10 years at diagnosis and 4% are younger than 5 years [2-5]. International epidemiologic data on CD vary considerably concerning the country and calendar year, and temporal trends are also controversial [1,6]. With an annual incidence of IBD of 5 to 11 per 100,000 children and adolescents, pediatrics face growing problems [3,5,7]. The incidence of CD is significantly higher compared to UC [1,8]; therefore, the following will focus on CD.

The etiology of CD is still not definitively understood. However, the etiology seems to be multifactorial and consists of an interaction of genetic, environmental, and lifestyle factors [9-11]. For IBD, the Western lifestyle has been discussed as the cause of CD for some time [12,13]. A similar international progression of incidence has also been observed for other immune-mediated chronic diseases, and inferences have been made about the influence of the Western lifestyle as measured by socioeconomic factors. For example, in their meta-analysis of diabetic ketoacidosis in type 1 diabetes, Große et al [14] identified an association between incidence and geographic as well as socioeconomic factors. Several studies also reported variations between incidence and geographic latitude for IBD [10,15]. The increase in CD incidence with latitude supports the hypothesis that higher residential sun exposure is associated with a lower risk of IBD. The results of these studies have been interpreted to suggest that low vitamin D status may be a risk factor for IBD [16]. The prevalence of vitamin D deficiency is global. Available data suggest that it occurs regardless of the development of the respective countries or the geographic latitude. Accordingly, consistent evidence indicates that the prevalence of vitamin D deficiency is highest in Asia, the Middle East, Africa, and countries with higher latitudes [17,18]. The medical and health-economic relevance of treating children and adolescents with IBD continues and is based on observations in several international studies, with the result that the number of pediatric IBD has increased and the onset of the disease seems to be shifting to early childhood. The impact of this shift in new cases is associated with a high individual as well as the societal burden of disease and will place a heavy burden on the respective health care systems [5,13,19,20]. This study aims to describe global trends in the incidence of CD since 1970 and to identify possible factors influencing the increasing incidence.

## Methods

### Study Design

A systematic review was conducted for IBD disorders. Studies were initially included from 1970 to 2019. To improve transparency in methodology, the study protocol for this review was published as “Study Protocol Epidemiology of Inflammatory Bowel Disease in Childhood and Adolescence: a Systematic Review” [21].

This systematic literature search was performed in the PubMed and Embase databases via Ovid. In addition, a manual search was performed in bibliographies of previously published and relevant systematic reviews. For detailed methodology and screening of this systematic review, we refer to the published study protocol [21].

For this study, we updated the previous systematic review from 2019 and 2020 to 2022. In this update, which was carried out until August 2022, we used the same search term as before but also included studies published up to December 31, 2021, which covered the observation period from 1970 to 2019. The complete search strategy can be viewed in Multimedia Appendix 1. The inclusion and exclusion criteria shown in Table 1 were defined for this study.

**Table 1:** Inclusion and exclusion criteria for this systematic review and meta-analysis according to the PICOS^a^ scheme.

	Inclusion criteria	Exclusion criteria
Population	Children and adolescents aged 0-18 years	Aged >18 years
Intervention	Incidence	No incidence
Comparison	Geographical characteristics, environmental factors, and economic factors	—^b^
Outcome	CD^c^	No IBD^d^, UC^e^, or IBD-U^f^ studies with unreported diagnostic criteria
Type of study	Cohort studies and register studies, prevalence studies and cross-sectional studies (population based)	Case-control studies, systematic reviews, meta-analyses, and case studies
Language	English, French, and German	All others

^a^PICOS: Population, Intervention, Comparison, Outcome, Study design.

^b^Not available.

^c^CD: Crohn disease.

^d^IBD: inflammatory bowel disease.

^e^UC: ulcerative colitis.

^f^IBD-U: indeterminate colitis.

The title and abstract screening and the full-text screening were carried out independently by 2 project participants. The extraction of the data and the corresponding consistency checks were also carried out by 2 project participants. In case of disagreement, a third project participant was consulted for mediation. All included studies were evaluated for study quality. The critical appraisal tools Critical Assessment of Structure Prediction and Scottish Intercollegiate Guidelines Network were used for this purpose. In addition, a risk of bias analysis, following the procedure described in the Cochrane Handbook [22], was performed (see Multimedia Appendix 2 [5-7,23-92]). Studies of poor quality were not excluded from the quantitative synthesis to avoid loss of information.

### Ethical Considerations

An ethics vote was not required for this systematic review because patients were not directly involved in this study.

### Data Extraction

All included studies were independently screened for incidence rates and study characteristics using a standardized table summary of findings. In case of missing data, contact with the authors was made. The data were exported to a database and processed for statistical analysis. For studies by 1 author that reported multiple incidence rates for children and adolescents, the mean values of incidence rates and study sizes were calculated for the respective observation period.

For the planned meta-regression, we classified possible moderators of heterogeneity into 2 dimensions: geographic and environmental factors on the one hand, and socioeconomic factors on the other. Longitude and latitude as well as exposure to UV sunlight (UV radiation index [UVI]) were assigned to the first dimension of geographical and environmental factors. The geographic data were extracted from Geoplaner (version 3.1; Martin Nathensen). When studies are nationwide or involve multiple centers within a country, the mean latitude value applied to the corresponding country or area was considered. In addition, we used the mean latitude to calculate the absolute distance to the equator irrespective of the northern or southern location [15]. We extracted the UVI from the United Nations Sustainable Development Goals data from the World Health Organization database [93].

The second dimension of possible moderators included socioeconomic factors. For this purpose, we used the percentage of gross domestic product (GDP) spent on health, which we extracted from the Organisation for Economic Co-operation and Development database “Health expenditure and financing” [94]. The Human Development Index (HDI) was included in the analysis as another possible moderator. The HDI assesses a country’s developmental state and combines life expectancy at birth, expected years of schooling, and gross national income per capita [95]. The values relevant to this study were extracted from the United Nations Development Programme’s Human Development Reports [95] from 1990 onward and averaged for statistical analysis. In addition, data on the GDP of the respective included countries from the GENESIS database of the Federal Statistical Office were used for further moderator analysis [96]. Furthermore, the universal health coverage (UHC) service coverage index Sustainable Development Goals 3.8.1 was extracted from the World Health Organization database [93]. UHC quantifies coverage of essential health services and is defined as the average coverage based on tracer interventions that include reproductive, maternal, newborn, child health, infectious diseases, noncommunicable diseases, service capacity, and access among the population [97].

### Statistical Analysis

We performed random-effects meta-analyses and meta-regressions to assess the variability of incidence rates. Analysis was performed with R (version 4.2.1.; R Foundation for Statistical Computing) software using the *metafor* package version 3.8-1 [98]. Meta-analysis was performed on a log scale (log incidence rates) using the general inverse variance method. Random effects and the extent of heterogeneity were estimated using the restricted maximum likelihood estimator. For the meta-regression, a multivariate model was constructed to identify further moderators of heterogeneity in incidence rates. The pooled incidence rates for each observation period formed the dependent variable. The observation period for each study was averaged and assigned as the starting time of the given study. The absolute distance of the included countries from the equator, UVI, HDI, health expenditure as a percentage of GDP, GDP, and UHC were included as additional independent variables in the regression model. In addition to the estimate of τ^2^, the Q-test for heterogeneity and the *I*^2^ statistic are reported. The *I*^2^ value was interpreted according to Higgins and Thompson [99] as follows: 0% to 40%, possibly insignificant; 30% to 60%, moderate heterogeneity; 50% to 90%, substantial heterogeneity; and 75% to 100%, considerable heterogeneity [99]. The influence of the moderators was evaluated using the *R*^2^ statistic as a measure of the explained heterogeneity. An a priori significance level of 0.05 (5%) was set for all statistical methods. To control the risk of publication bias, statistical methods such as the Egger regression test and the rank correlation test were applied to quantitatively assess the risk of publication bias. In addition, we applied the trim-and-fill analysis and the fail-safe N-analysis (Rosenberg method) to consider and control the potential risk of publication bias.

## Results

### Data Basis and General Assessment of Studies

**Figure 1:**
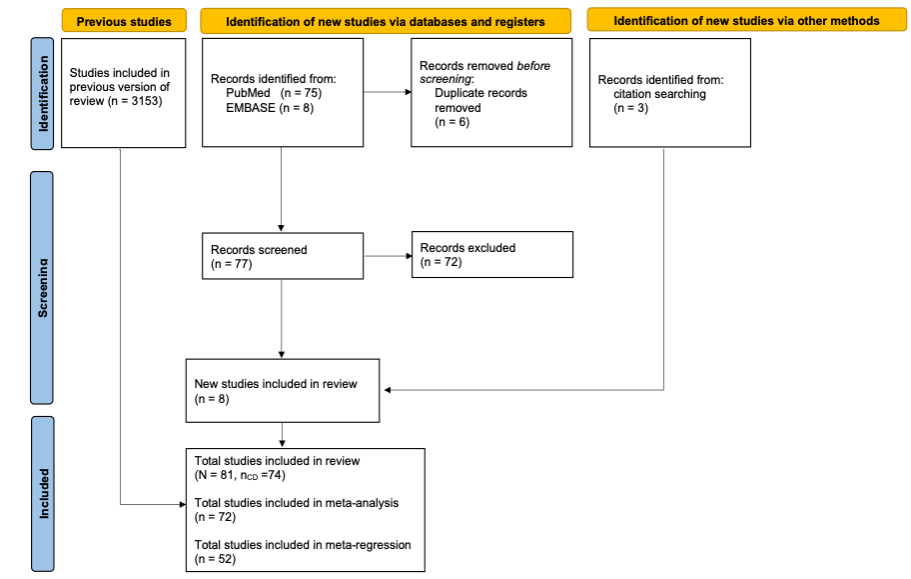
PRISMA 2020 flow diagram for updated systematic reviews which included searches of databases, registers, and other sources, adapted from Page et al, with permission from PRISMA, for our systematic review with meta-analysis and meta-regression [100]. PRISMA: Preferred Reporting Items for Systematic Reviews and Meta-Analyses.

A total of 3153 studies were found from the previous systematic search conducted in 2019. The update of the systematic literature search yielded another 83 records. After removing duplicates, 77 studies were screened in the systematic literature search update. Another 5 studies from the update were included in the qualitative and quantitative synthesis. In total, the systematic literature research resulted in 81 findings from 29 countries with the search terms CD, UC, and indeterminate colitis. CD was the subject of a total of 74 studies, which were included in the qualitative synthesis of this work. Further, 2 studies had to be excluded retrospectively due to the lack of population reference. The meta-analysis included 72 studies from 26 countries and the meta-regression included 52 studies (Figure 1 [100]). The references of the included studies and a table summary of included studies can be viewed in Multimedia Appendices 3 [6,7,23-75,77-92] and 4 [7,23-75,77-92,101].

In the 72 studies eligible for meta-analysis, the incidence rates on the linear scale varied from 0.14/10^5^ to 11.40/10^5^. Performing a random-effects meta-analysis revealed that the overall mean incidence rate was 2.64/10^5^ (95% CI 2.09 to 3.34; on log-scale –10.54, 95% CI –10.78 to –10.31), whereas the *I*^2^ value of 97.88% suggests that the substantial heterogeneity of this study’s results cannot be explained by a sampling effect alone (Multimedia Appendix 5 [5-7,23-92]). In the following, we set out to identify factors that can account for the substantial dispersion in study results. Interestingly, the individual weights for each study were largely dominated by the contribution of the between-study variance while the study-specific variance (ie, the sampling effect) had a smaller effect. Consequently, the studies in the random-effects meta-analysis have rather similar relative weights.

### Time as a Moderator of CD Incidence

**Figure 2:**
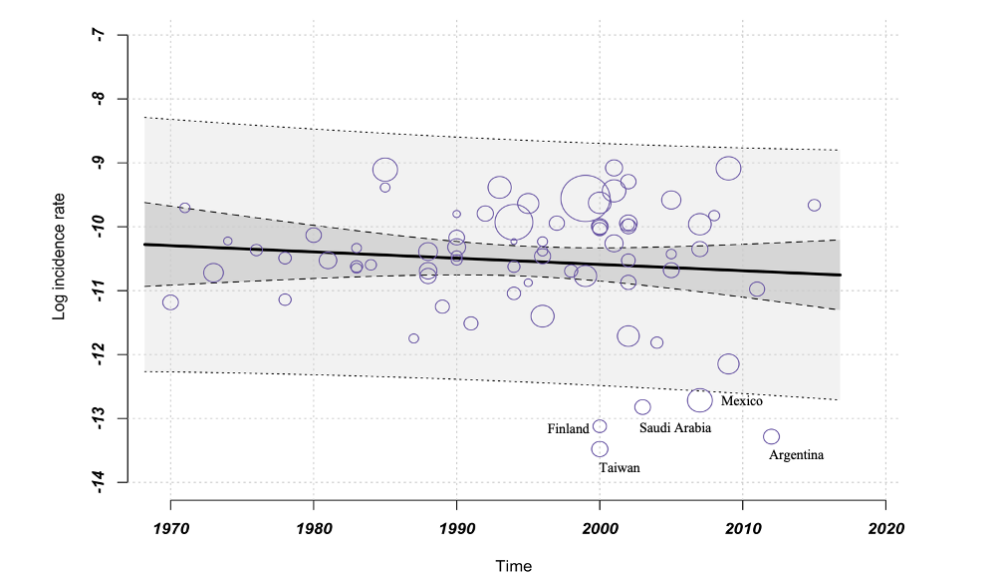
Meta-regression: incidence over time: dependent variable incidence CD, independent variable time, bubbles symbolize the studies that have been included, with each bubble’s size corresponding to the weighting assigned to the respective study (k=72, estimator: REML): test of moderators F test=0.71; *P*=.39; I2=97.85%; variance explanation via R2 0%. CD: Crohn disease; REML: restricted maximum likelihood.

We included studies from a 50-year observation period, from 1970 to 2019. To assess whether the time point of this study influenced the CD incidence, we performed a meta-regression in which *time* is considered the continuous variable, whereas *incidence rates* are the dependent variable. Figure 2 confirms that the moderator *time* has no significant effect on incidence rates for CD. Moreover, *time* as a moderator cannot explain the heterogeneity, so the remaining heterogeneity remains substantial (test of moderators *P*=.39; *I*^2^=97.85%; *R*^2^=0.00; see also Multimedia Appendix 6-11 [5-7,23-92] subgroup analysis incidence CD in 10-year steps). These results suggest that there must be other moderators to explain the observed heterogeneity. Figure 2 also displays a slight negative trend with a simultaneous increase in heterogeneity. Some of the studies with low incidence values (depicted in the lower right corner) are from Taiwan, Finland, Saudi Arabia, Mexico, and Argentina, reinforcing the impression of greater geographical division. In the next step, we specifically examine the influence of the geographic component on CD incidence rates.

### Geographical and Environmental Factors as Moderators of CD Incidence Rates

**Figure 3:**
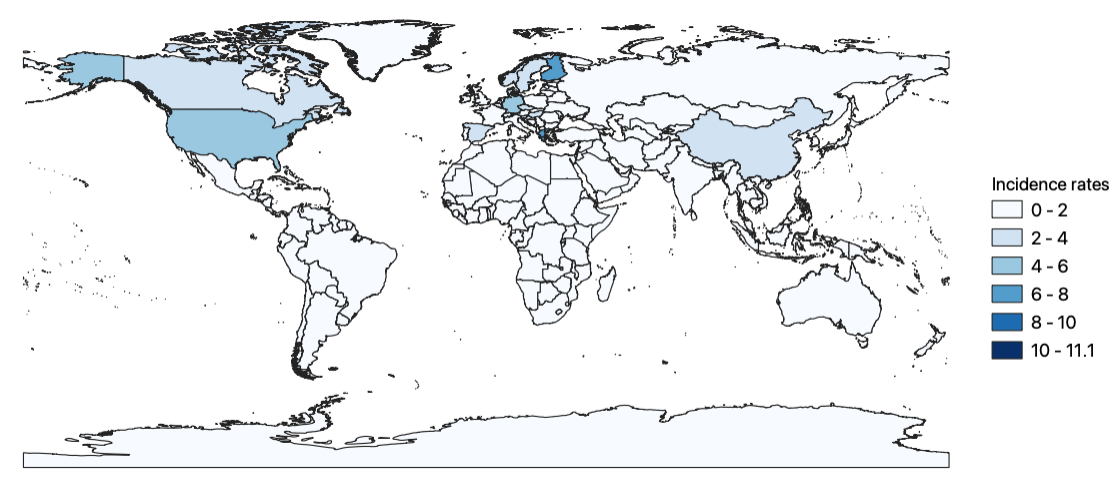
Geographical distribution of Crohn disease incidence (raw data).

It is interesting to see that the highest mean incidence rates per 100,000 children and adolescents during the observation period from 1970 to 2019 were observed in Australia (11.12 new cases/10^5^), Finland (6.31/10^5^), Canada (7.12/10^5^), Germany (6.15/10^5^), and New Zealand (6.07/10^5^). The lowest incidence rates were reported in studies from countries in Asia and South America. Strikingly, an incidence of CD almost twice as high was reported in Australia compared to the other included countries (Figure 3 and forest plot geographic variation in incidence rates of CD in Multimedia Appendix 12). These data suggest geographic heterogeneity, which we first consider at the continental level.

Indeed, a meta*-*regression with the variable *continents* indicated that 41.34% of the heterogeneity can be explained. The test for moderators is significant (*P*<.001). Comparing this regression model with a more complex model in which we added the factor *time* to the moderator *continents*, an ANOVA showed no significant model improvement, confirming the notion that time does not act as a major moderator. In a further step, we examined the development of the incidence of CD over time for each continent individually. The results of this analysis suggest that the incidence of CD has developed differently in relation to the continents. Increasing trends were noted for North America, Europe, and Asia. For South America and Australia or the Pacific region, we found no confirmation of increasing incidence because of too few data points (see Multimedia Appendices 13 and 14).

**Figure 4:**
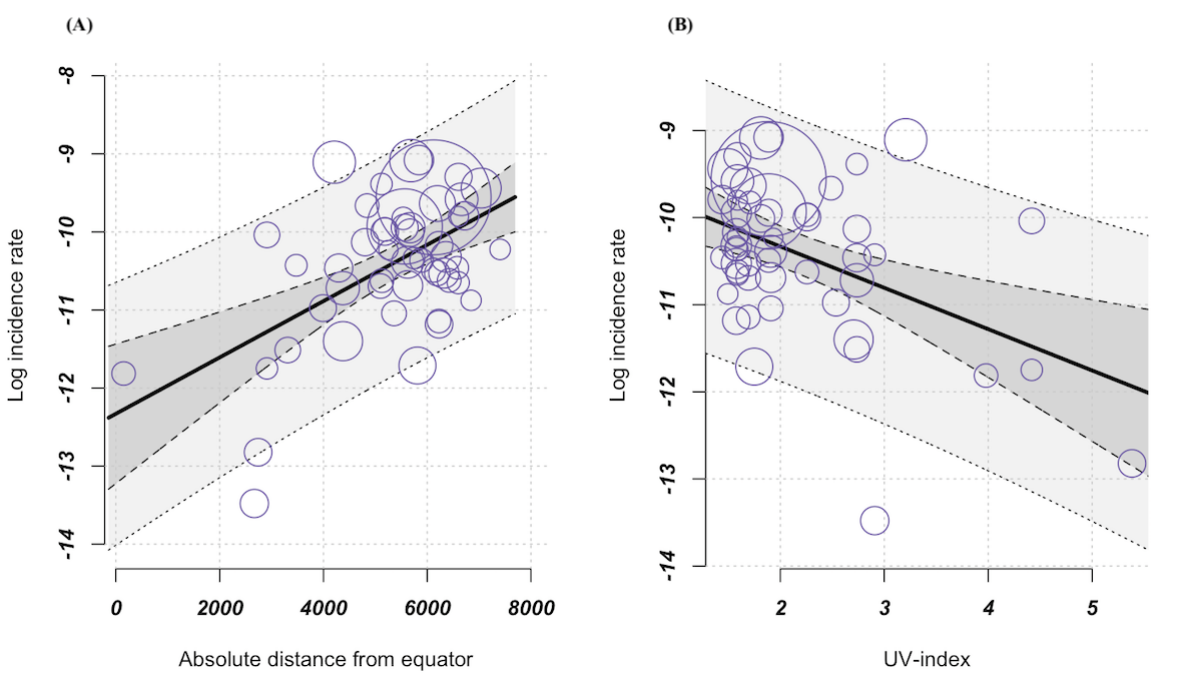
Meta-regression, (A) increasing incidence with increasing distance from the equator; variable incidence CD, independent variable absolute distance from the equator (k=52, estimator: REML): test of moderators F test=18.78; *P*<.001; I2=96.29%; variance explanation via R2 29.14%. (B) Decreasing incidence with increasing UVI; variable incidence CD, independent variable UVI (k=52, estimator: REML): test of moderators F test=11.35; *P*<.001; I2=96.94%; variance explanation via R2 18.57%; bubbles symbolize the studies that have been included, with each bubble’s size corresponding to the weighting assigned to the respective study. CD: Crohn disease; REML: restricted maximum likelihood; UV: ultraviolet; UVI: ultraviolet radiation index.

The results also suggest that distance from the equator may affect the incidence of CD. A corresponding meta-regression, which included *absolute distance from the equator* as a moderator, showed that CD incidence increased significantly with increasing distance from the equator (Figure 4). Extrapolated to 1000 km, the incidence rate increased by 0.36%. The test for moderators yielded a significant result (*P*<.001). However, given the considerable heterogeneity in study results, distance from the equator formally contributed only moderately to better explain this variance (*R*^2^=29.14%; Table 1 and Figure 4). We found similar results when we recalculated the analysis for the *country-specific* UVI instead of the *absolute distance from the equator*. The results show that incidence rates decrease with increasing UV exposure. The results with this factor were significant in the test of moderators (*P*<.001) and 18.57% heterogeneity was resolved (Table 1 and Figure 4). Given the correlation between the moderators of absolute distance from the equator and UVI (r=–0.87, *P*<.001), we refrained from a joint regression model to avoid problems of collinearity and unreliable coefficient estimates.

### Socioeconomic Factors as Moderators of CD Incidence Rates

**Figure 5:**
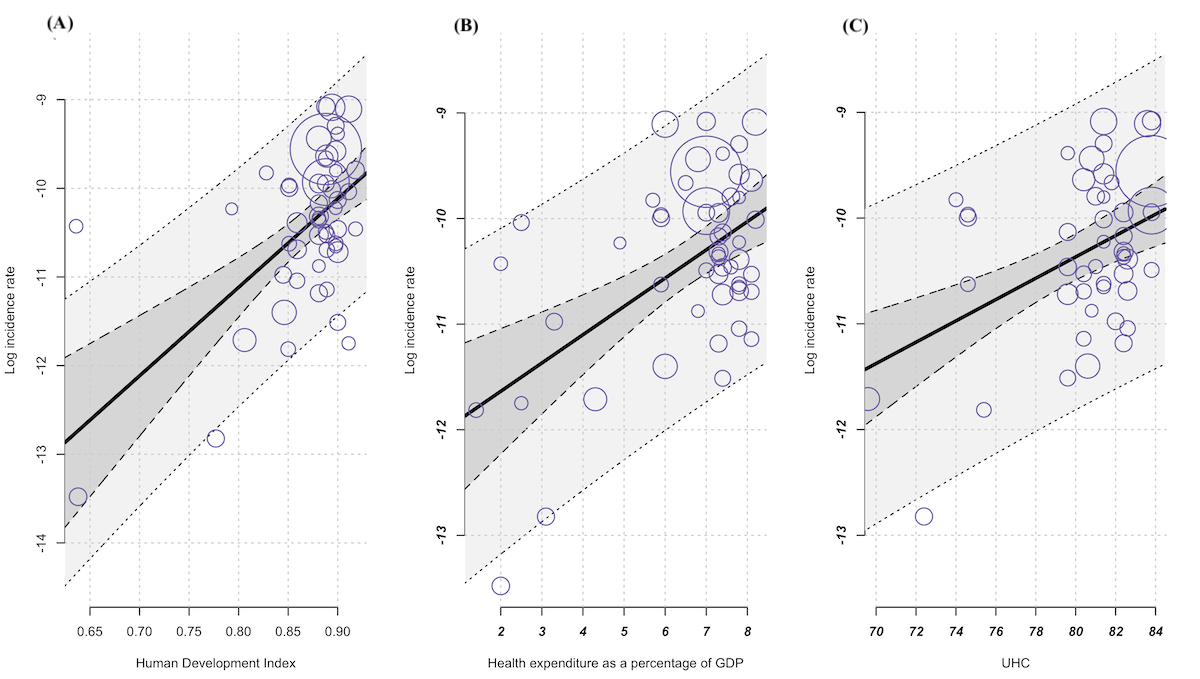
Meta-regression: (A) increasing incidence with increasing HDI; variable incidence CD, independent variable HDI (k=52, estimator: REML): test of moderators F test=26.4; *P*<.001; I2=95.87%; variance explanation via R2=40.8%. (B) Increasing incidence with increasing CHE-GDP%; variable incidence CD, independent variable CHE-GDP% (k=52, estimator: REML): test of moderators F test=18.78; *P*<.001; I2=96.53%; variance explanation via R2=29.4%. (C) Increasing incidence with increasing universal health coverage index SDG 3.8.1; variable incidence CD, independent variable universal health coverage (k=52, estimator: REML): test of moderators F test=17.27; *P*<.001; I2=96.33%; variance explanation via R2=28.86; bubbles symbolize the studies that have been included, with each bubble’s size corresponding to the weighting assigned to the respective study. CD: Crohn disease; CHE-GDP%: health expenditure as a percentage of gross domestic product; HDI: Human Development Index; REML: restricted maximum likelihood; UHC: universal health coverage; SDG: Sustainable Development Goals.

In the next step, we investigated the extent to which socioeconomic factors could be considered moderators of heterogeneity. The results of the corresponding meta-regression showed that the *HDI*, *health expenditure* as a percent of GDP, and the UHC *index* acted as moderators. Accordingly, the frequency of CD increases with increasing values of each moderator (Table 2 and Figure 5). To avoid issues with collinearity and unreliable coefficient estimates resulting from the correlations between the socioeconomic factors, we decided not to use a joint regression model.

**Table 2:** Meta-regression results. Dependent variable: incidences CD^a^; independent variables: absolute distance to the equator and UVI^b^. ME^c^ model (k=52, estimator: REML^d^).

Moderator	Estimate	SE	*z* value	*P* value	95% CI	*I*^2^ (%)	*R*^2^ (%)
Absolute distance to the equator	0.0003	0.45	–27.06	<.001	0.0001 to 0.0005	96.29	29.14
UVI	–0.474	0.141	–3.37	<.001	–0.75 to –0.19	96.94	18.57

^a^CD: Crohn disease.

^b^UVI: ultraviolet radiation index.

^c^ME: mixed effects.

^d^REML: restricted maximum likelihood.

### Multifactorial Regression Model to Explain CD Incidence Rates

In our analysis, we identified different widely independent factors *study timing*, *absolute distance from the equator*, and *HDI* or UHC as univariate moderators of CD incidence rates. To explain the high degree of heterogeneity between studies that we observed during the analyzed study period, we used a multifactorial meta-regression model that accounted for these complementary moderators as the final step of our investigation. As a result, the corresponding model showed a joint *R*^2^ of 62.5%, indicating that almost two-thirds of the heterogeneity can be explained by these 3 moderators. The test for moderators was significant at *P*<.001 (see Tables 3 and 4).

**Table 3:** Meta-regression results. Fependent variable: incidences CD^a^; independent variables: HDI^b^, CHE-GDP%^c^, GDP^d^, and UHC^e^ service coverage index SDG^f^ 3.8.1. ME^g^ model (k=52, estimator: REML^h^).

Moderator	Estimate	SE	*z* value	*P* value	95% CI	*I*^2^ (%)	*R*^2^ (%)
HDI	9.98	1.94	5.14	<.001^i^	6.18 to 13.79	95.87	40.8
CHE-GDP%	0.26	0.06	4.33	<.001^i^	0.15 to 0.39	96.53	29.4
GDP	<0.00	0.0004	–0.02	.98	–0.0001 to 0.0001	97.57	<0.00
Universal health coverage	0.1	0.02	4.12	<.001^i^	0.05 to 0.15	96.33	28.86

^a^CD: Crohn disease.

^b^HDI: Human Development Index.

^c^CHE-GDP%: health expenditure as a percentage of gross domestic product.

^d^GDP: gross domestic product.

^e^UHC: universal health coverage.

^f^SDG: Sustainable Development Goals.

^g^ME: mixed effects.

^h^REML: restricted maximum likelihood.

^i^significant.

**Table 4:** Meta-regression results. Dependent variable: incidences CD^a^; independent variables: time, absolute distance from equator, and Human Development Index (HDI^b^)/ ME^c^ model (k=52, estimator: REML^d^). Test of moderators F test=24.57; *P*<.001^e^.

Moderator	Estimate	SE	*z* value	*P* value	95% CI	*I*^2^ (%)	*R*^2^ (%)
Time	0.030	0.01	3.48	<.001	0.013 to 0.047	92.99	62.56
Absolute distance to the equator	0.0003	0.0001	3.69	<.001	0.0001 to 0.0004	92.99	62.56
HDI	9.50	1.84	5.15	<.001	1.914 to 10.153	92.99	62.56

^a^CD: Crohn disease.

^b^HDI: Human Development Index.

^c^ME: mixed effects.

^d^REML: restricted maximum likelihood.

^e^multifactorial model: incidence rate ~(time + absolute distance to the equator + Human Development Index).

## Discussion

Our systematic review with meta-analysis and meta-regression examined global trends in the incidence of CD. Although several individual studies reported an increase in incidence rates for CD in a certain (national) cohort, few high-quality studies were able to substantiate and quantify such an increase (risk of bias analysis in Multimedia Appendix 2). Furthermore, some of the studies reporting temporal trends in CD incidence rates were controversially discussed [1,6]. Different study designs also made it difficult to compare incidence rates over time, which may further contribute to the substantial heterogeneity in incidence rates.

In this systematic review, we evaluated a total of 72 studies from 26 countries on the incidence of CD over a 50-year observation period. We found substantial heterogeneity in incidence rates, which was confirmed by meta-analysis using a random effects model (Cochrane Q=3349.38; *P*<.001; *I*^2^=97.88%). Despite the large heterogeneity of the data, we obtained several interesting results. First, we found no clear evidence of a general global trend toward increasing CD incidence rates over time. While incidence rates might increase within individual countries, it rather appears that the inclusion of studies from a broader range of countries also increases the overall between-study heterogeneity to the extent that a global temporal trend is not identifiable. While incidence rates might increase within individual countries, it rather appears that the inclusion of studies from a broader range of countries also increases the overall between-study heterogeneity to the extent that a global temporal trend is not identifiable. This might be a result of differences in methodology and how individual studies reported incidence rates over time and needs further investigation in future research. The fact that little data were available in certain regions may also have contributed to the fact that a global trend over time was not discernible from our analyses. Kuenzig et al [102] reported similar issues in this regard. Due to the different reporting of incidence rates of IBD in childhood and adolescence, they also had difficulties in describing a clear temporal trend. Second, we observed a dependency of the incidence rates on the geographic location, with increasing incidence for countries that are further away from the equator. Third, we observed a similar effect for several socioeconomic factors, in which higher scores correlated significantly with higher CD incidence rates.

Regarding the geographic differences in incidence rates of CD, several studies reported a north-south gradient. For example, Nerich et al [15] reported the effects of latitude on the geographic distribution of CD. However, quantification of the gradient by latitude was not performed. Armitage et al [23] similarly reported a significant north-south gradient for CD in Scotland. Since recent epidemiologic studies have reported an increasing incidence of IBD worldwide, including in southern countries, particularly also in the southern hemisphere [2,103], we decided to use absolute distance from the equator as a factor to represent and quantify a relationship between incidence and geographic location. The result of our meta-regression showed that incidence rates increased with increasing distance from the equator. This result corresponds with the results of our further analysis of possible moderators of incidence rates. We found that countries with a high UVI, other than Australia, have a lower incidence of CD. Our results correlate with findings from other studies showing that higher exposure to UV radiation, or sunlight, is associated with a lower risk of CD and IBD [16,104]. In our results, Australia stood out with a high incidence. Although Australia is considered sun-rich, 17% of Australian adolescents have vitamin D deficiency [104]. Vitamin D is formed in the skin when exposed to UV radiation. We therefore suspect a correlation between vitamin D status and the incidence of CD. Further studies should therefore examine in particular whether a low vitamin D status is a risk factor for CD or IBD or a consequence. However, studies show that patients with IBD and especially CD also show a low vitamin D status and indicate a correlation with disease activity. Unfortunately, disentangling causality and correlation is an unresolved challenge in the ongoing debate about the interplay between vitamin D and IBD [105-107].

Although latitude or absolute distance from the equator and UV exposure are correlated, they cannot be fully replaced in our statistical analysis.

Our results concerning the socioeconomic factors contribute to the hypothesis that CD might correlate with industrialized, urbanized societies, largely due to a Western lifestyle and other associated environmental factors [13] which themselves go along with higher socioeconomic scores. It is also known that the incidence and prevalence of CD vary between countries with different HDIs [108-110]. Although there are few epidemiologic studies of CD in low- and middle-income countries, the incidence of CD is increasing significantly worldwide, affecting even countries previously considered to be at low risk [103,111]. It has been observed that the incidence and prevalence in middle-income countries are also increasing in children and adolescents, which has been attributed to the rapid modernization and Westernization of the population [13]. Our findings seem to follow a global pattern, namely that the process of industrialization has an impact on the incidence of CD. In this regard, we also follow the view of Takahashi et al [109] and Ananthakrishnan et al [103] that the level of development of countries and Western lifestyles are related to the level of incidence. However, causality cannot be inferred from our study. Further research is needed for this purpose. In recent times, there has been a growing significance attributed to observational research conducted on real-world data, leading to the establishment of global research networks, exemplified by the Observational Health Data Sciences and Informatics community. These networks aim to facilitate large-scale studies grounded in the Observational Medical Outcomes Partnership (OMOP) common data model (CDM). Consequently, the use of observational studies that use real-world data is a valuable way to study CD and IBD in the future [112].

### Limitations

This study is limited by the use of 2 databases for systematic literature search. Another limitation of this study is the exclusion of studies that were not published in English, Spanish, French, or German. Given that the included studies were mainly from countries with good access to the health care system (UHC >70), the underreporting of countries with poorer access to the health care system should be discussed. We controlled the risk of publication bias using the Eggers regression test, rank correlation test, trim and fill analysis, and fail-safe N analysis (Rosenberg method). Although these methods did not statistically indicate a bias due to publication bias, a small bias cannot be completely ruled out.

### Conclusions

Based on available study data from 1970 to 2019, we could not identify a global, temporal trend toward increasing CD incidence rates, although these effects are reported for individual countries or regions. Instead, we could demonstrate that a substantial part of the observed heterogeneity between the published study results can be explained by geographic location and socioeconomic factors. Our study can be used to provide quantitative estimates of these trends for CD in childhood and adolescence. However, to establish causal relationships regarding potential risk factors, further studies are necessary, including those conducted in countries with lower levels of development. Nevertheless, our analysis provides valuable information to drive future research and health policies aiming to reduce the incidence of CD among children and adolescents. This needs continuous global monitoring of the incidence of IBD in childhood and adolescence to fully understand the trends in IBD incidence [102]. To this end, the implementation of internationally standardized and interoperable registries, coupled with the dissemination of health data via federated networks grounded on a CDM, such as the OMOP CDM, is deemed advantageous. The OMOP CDM aligns most closely with the requisites conducive to expediting data exchange within longitudinal studies [112,113]. The usage of such registries and data networks holds the potential to streamline the exhaustive and standardized accumulation as well as dissemination of data. This, in turn, would enhance our comprehension of CD and foster evidence-based approaches for preventive and interventional strategies.

## Appendix

Multimedia Appendix 1 Search strategy systematic literature search in Pubmed and Embase via Ovid.

Multimedia Appendix 2 Risk of bias analysis.

Multimedia Appendix 3 Summary of the included studies.

Multimedia Appendix 4 References of the found studies.

Multimedia Appendix 5 Forest plot meta-analysis across all studies on the log scale; random effects model (k=72; τ2 estimator: REML [restricted maximum likelihood]).

Multimedia Appendix 6 Subgroup analysis incidence CD (Crohn disease) in 10-year steps: forest plot pooled incidence rates per 10 years; random effects model (k=5; τ2 estimator: REML [restricted maximum likelihood]).

Multimedia Appendix 7 Subgroup analysis incidence CD (Crohn disease) in 10-year steps: forest plot studies period 1970-1979; random effects model (k=8; τ2 estimator: REML [restricted maximum likelihood]).

Multimedia Appendix 8 Subgroup analysis incidence CD (Crohn disease) in 10-year steps: forest plot studies period 1980-1989; random effects model (k=16; τ2 estimator: REML [restricted maximum likelihood]).

Multimedia Appendix 9 Subgroup analysis incidence CD (Crohn disease) in 10-year steps: forest plot studies period 1990-1999; random effects model (k=25; τ2 estimator: REML [restricted maximum likelihood]).

Multimedia Appendix 10 Subgroup analysis incidence CD (Crohn disease) in 10-year steps: forest plot studies period 2000-2009; random effects model (k=33; τ2 estimator: REML [restricted maximum likelihood]).

Multimedia Appendix 11 Subgroup analysis incidence CD (Crohn disease) in 10-year steps: subgroup analysis: forest plot studies period 2010-2019; random effects model (k=12; τ2 estimator: REML [restricted maximum likelihood]).

Multimedia Appendix 12 Geographic variation in incidence rates of CD (Crohn disease): forest plot pooled incidence rates CD by continent; random effects model (k=5; τ2 estimator: REML [restricted maximum likelihood]).

Multimedia Appendix 13 Meta-regression, subgroups continents, dependent variable incidence CD (Crohn disease), independent variable: time, bubbles symbolize the studies that have been included, with each bubble's size corresponding to the weighting assigned to the respective study.

Multimedia Appendix 14 Meta-regression results: dependent variable incidences CD (Crohn disease), independent variables: time, 1 too few observations for regression analysis, pooled IR (incidence rate) 0.23/105 (on log scale –12.96), a multiplicative change factor >1 increasing IR, <1 decreasing IR.

Multimedia Appendix 15 PRISMA (Preferred Reporting Items for Systematic Reviews and Meta-Analyses) checklist.

## Abbreviations

CD: Crohn disease
CDM: common data model
GDP: gross domestic product
HDI: Human Development Index
IBD: inflammatory bowel disease
OMOP: Observational Medical Outcomes Partnership
UC: ulcerative colitis
UHC: universal health coverage
UV: ultraviolet
UVI: ultraviolet radiation index

## Data Availability

The data sets generated and analyzed during this study are available from the corresponding author upon reasonable request.
