# Supplementary material for: Correlation of Socioeconomic and Environmental Factors With Incidence of Crohn Disease in Children and Adolescents: Systematic Review and Meta-Regression
Source: JMIR Public Health Surveill. 2024 Mar 25;10:e48682. doi: 10.2196/48682 (PMC11002755; doi:10.2196/48682)
Supplement: Multimedia Appendix 1 [file publichealth_v10i1e48682_app1.pdf]

1. Systematic literature search strategy

PubMed search strategy

1. colitis, ulcerative[MeSH Terms]
2. OR crohn disease[MeSH Terms]
3. OR Crohn Disease[Text Word
4. OR Crohn´s Disease[Text Word]
5. OR inflammatory bowel diseases[MeSH Terms]
6. OR Inflammatory Bowel Disease*[Text Word]
7. AND
8. (incidence[MeSH Terms]
9. OR incidence[Text Word]
10. OR prevalence[MeSH Terms]
11. OR prevalence[Text Word]
12. AND
13. 1970:2019[pdat]
14. AND
15. "humans"[MeSH Terms]
16. AND
17. "infant"[MeSH Terms]
18. OR "child"[MeSH Terms]
19. OR "adolescent"[MeSH Terms]
20. OR "infant"[MeSH Terms]
21. OR "infant"[MeSH Terms:noexp]
22. OR "child, preschool"[MeSH Terms]
23. OR "child"[MeSH Terms:noexp]
24. OR "adolescent"[MeSH Terms]

EMBASE via Ovid search strategy

1. colitis, ulcerative.mp or exp ulcerative colitis/
2. crohn disease.mp or exp Crohn disease/
3. Inflammatory Bowel Disease.mp or exp inflammatory bowel disease/
4. exp standardized incidence ratio/ and incidence.mp and exp incidence/
5. prevalence.mp. or exp prevalence/
6. 1 or 2 or 3
7. 4 and 6
8. 4 or 5
9. limit 8 to (human and yr="1970 - 2019" and (infant <to one year> or child <unspecified age> or preschool child <1 to 6 years> or school child <7 to 12 years> or adolescent <13 to 17 years>))
