# Supplementary material for: Correlation of Socioeconomic and Environmental Factors With Incidence of Crohn Disease in Children and Adolescents: Systematic Review and Meta-Regression
Source: JMIR Public Health Surveill. 2024 Mar 25;10:e48682. doi: 10.2196/48682 (PMC11002755; doi:10.2196/48682)
Supplement: Multimedia Appendix 2 [file publichealth_v10i1e48682_app2.pdf]

Risk of Bias

Author	Recruitment procedere & follow up	Outcome definition und measurement	Outcome source and validation	Confounding and effect modification	Analysis method: methods to reduce research specific	Funding	Conflikt of Interesst
Abramson et al. (2010)	🟢	🟢	🟢	🟢	🟢	🟢	🟢
Adamiak et al. (2013)	🟢	🟢	🟢	🟢	🟢	🟢	🟢
Ahmed et al. (2006)	🟢	🟢	🟢	🟡	🟡	🟢	🟢
Armitage et al. (2001)	🟢	🟢	🟢	🟡	🟡	🟢	🟢
Ashton et al. (2014)	🟢	🟢	🔴	🔴	🟡	🟢	🟢
Auvin et al. (2005)	🟢	🟢	🟢	🟢	🟢	🟢	🟢
Barton et al. (1989)	🟢	🟢	🟢	🟡	🟢	🟢	🟢
Benchimol et al. (2014)	🟢	🟢	🟢	🟢	🟢	🟢	🟢
Benchimol et al. (2017)	🟢	🟢	🟢	🟢	🟢	🟢	🟢
Bentsen et al. (2002)	🟢	🟢	🟢	🟡	🟡	🟢	🟢
Bitton et al. (2014)	🟢	🟢	🔴	🔴	🟡	🟢	🟢
Calkins et al. (1984)	🟢	🟡	🟡	🟡	🟡	🟢	🟢
Cosgrove et al. (1996)	🟢	🟢	🟡	🔴	🟡	🟢	🟢
El Mouzan et al. (2014)	🟢	🟢	🟢	🟢	🟢	🟢	🟢
El-Matary et al. (2014)	🟢	🟢	🟢	🟢	🟢	🟢	🟢
Fellows et al. (1990)	🟢	🟢	🟢	🟢	🟢	🟢	🟢
Ghione et al. (2018)	🟢	🟢	🟡	🟡	🟢	🟢	🟢
Gottrand et al. (1991)	🟢	🟢	🟡	🟢	🟢	🟢	🟢
Grieci, Büttner (2009)	🟢	🟢	🟢	🟢	🟢	🟢	🟢
Haug et al. (1989)	🟢	🟢	🟢	🟡	🟢	🟢	🟢
Henderson et al. (2012)	🟢	🟢	🟢	🟢	🟢	🟢	🟢
Hildebrand et al. (1994)	🟢	🟢	🟢	🟡	🟡	🟢	🟢
Hildebrand et al. (2003)	🟢	🟢	🟡	🔴	🟢	🟢	🟢
Hong et al. (2018)	🟢	🟢	🟢	🟢	🟢	🟢	🟢
Hope et al. (2012)	🟢	🟢	🟢	🟢	🟢	🟡	🟡

Risk of Bias

Author	Recruitment procedere & follow up	Outcome definition und measurement	Outcome source and validation	Confounding and effect modification	Analysis method: methods to reduce research specific bias	Funding	Conflikt of Interesst
Isa et. Al. (2018)	🟡	🟢	🟡	🟡	🔴	🟢	🟢
Jacobsen B.A. et al. (2006)	🟢	🟢	🟡	🟡	🟢	🟢	🟢
Jacobsen, C. et al. (2008)	🟢	🟢	🟡	🟡	🟡	🟢	🟢
Jacobsen, C. et al. (2009)	🟢	🟢	🟢	🟢	🟢	🟢	🟢
Jacobsen, C. et al. (2011)	🟡	🟢	🟢	🔴	🔴	🟢	🟢
Jussila et al. (2012)	🟢	🟢	🟡	🟡	🟢	🟢	🟢
Karolewska-Bochenek et al. (2009)	🟢	🟢	🟢	🟢	🟡	🟢	🟢
Kern et al. (2021)	🟢	🟢	🟢	🟢	🟢	🟢	🟢
Kugathasan et al. (2003)	🟡	🟢	🟢	🔴	🔴	🟢	🟢
Kuo et al. (2015)	🟢	🟢	🟡	🟡	🟢	🟢	🟢
Kwak et al. (2019)	🟢	🟢	🟢	🟢	🟡	🟢	🟢
Larsen et al. (2016)	🟢	🟢	🟡	🟡	🔴	🟢	🟢
Lethinen et al. (2011)	🟢	🟢	🟢	🟢	🟢	🟢	🟢
Lindberg et al. (1991)	🟢	🟢	🟢	🟢	🟡	🟢	🟢
Lindberg et al. (2000)	🟢	🟢	🟡	🟡	🟡	🟢	🟢
Lindquist et al. (1984)	🟢	🟢	🟢	🟢	🟢	🟢	🟢
Lopez et al. (2018a)	🟢	🟢	🟢	🟡	🟡	🟢	🟢
Lopez et al.(2018)	🟢	🟢	🟡	🟡	🟡	🟢	🟢
Lovasz et al. (2014)	🟢	🟢	🟢	🟢	🟡	🟢	🟢
Malaty et al. (2010)	🟡	🟡	🟡	🔴	🟡	🟢	🟢
Malmborg et al. (2013)	🟢	🟢	🟢	🟡	🟡	🟢	🟢
Martin-de -Carpi et al. (2013)	🟡	🟡	🟡	🔴	🟡	🟢	🟢
Muller et al. (2013)	🟢	🟢	🟡	🔴	🟡	🟢	🟢
Nyhlin et al. (1986)	🟢	🟡	🟡	🔴	🔴	🟡	🟡
Olafsdottir et al. (1989)	🟢	🟢	🟢	🟢	🟢	🟡	🟢
Ong et al. (2018)	🟢	🟢	🟡	🟡	🟡	🟢	🟢

**Author**	**Recruitment procedere & follow up**	**Outcome definition und measurement**	**Outcome source and validation**	**Confounding and effect modification**	**Analysis method: methods to reduce research specific bias**	**Funding**	**Conflikt of Interesst**
Orel et al. (2009)	🟢	🟢	🟡	🟡	🟡	🔴	🟢
Phavichitr et al. (2003)	🟢	🟢	🟢	🟢	🟢	🟢	🟢
Schwarz et al. (2017)	🟢	🟢	🟢	🟡	🟡	🟢	🟢
Shivashankar et al. (2017)	🟢	🟢	🟢	🟢	🟢	🟢	🟢
Sjoberg et al. (2013)	🟢	🟢	🟢	🟡	🟢	🟡	🟢
Sjoberg et al (2014)	🟢	🟢	🟡	🔴	🟡	🟢	🟢
Stordal et al. (2004)	🟢	🟢	🟢	🟢	🟢	🟢	🟢
Stowe et al. (1990)	🟢	🟢	🟢	🟡	🟡	🟢	🟢
Turunen et al. (2006)	🟢	🟢	🟢	🟡	🟡	🟢	🟢
Urlep et al. (2014)	🟢	🟢	🟢	🟡	🟡	🟢	🟢
Urlep et al. (2015)	🟢	🟢	🟢	🟡	🟡	🟢	🟢
Urne et al. (2002)	🟢	🟢	🟢	🟡	🟡	🟡	🟢
van der Zaag-Loonen et al. (2004)	🟢	🟢	🟢	🟢	🟡	🟢	🟢
Vicentin et al. (2017)	🟢	🟢	🟢	🟢	🟢	🟡	🟡
Virta et al. (2017)	🟢	🟢	🟢	🟢	🟢	🟢	🟢
Wang et al. (2013)	🟢	🟢	🟡	🟡	🟡	🟢	🟢
Watson et al. (2002)	🟢	🟢	🟡	🔴	🟡	🟡	🟡
Wittig et al. (2019)	🟡	🟡	🟢	🟡	🟢	🟢	🟡
Yamamoto-Furusho et al. (2019)	🟢	🟢	🟢	🟡	🟢	🟢	🟢
Yap et al. (2008)	🟢	🟢	🟢	🟡	🟡	🟢	🟢
Zyyani et al. (2017)	🟢	🟢	🟢	🟡	🟡	🟢	🟢
