# Supplementary material for: Correlation of Socioeconomic and Environmental Factors With Incidence of Crohn Disease in Children and Adolescents: Systematic Review and Meta-Regression
Source: JMIR Public Health Surveill. 2024 Mar 25;10:e48682. doi: 10.2196/48682 (PMC11002755; doi:10.2196/48682)
Supplement: Multimedia Appendix 3 [file publichealth_v10i1e48682_app3.pdf]

Summary of the included studies

ID	Author	Year	Start	Cont	Pop	size	IR	lower	upper	SEM
1	Abramson et al. (2010)	2010	1996	North America	750000	675	2,86	2,07	3,65	0,38
2	Adamiak et al. (2013)	2013	2000	North America	1300000	675	6,6	.	.	.
3	Ahmed et al. (2006)	2006	1996	Europe	90000	992	3,6	.	.	.
4	Armitage et al. (2001)	2001	1981	Europe	1156631	652	2,68	1,8	3,55	0,42
5	Ashton et al. (2014)	2014	2002	Europe	650000	195397	4,79	1,69	7,9	0,98
6	Auvin et al. (2005)	2005	1988	Europe	1312141	7066	2,28	2,17	2,39	0,05
7	Barton et al. (1989	1989	1970	Europe	1363573	9688	1,39	1,13	1,65	0,13
8	Benchimol et al. (2014)	2014	1994	North America	13500000	2667	4,87	0,78	8,95	1,77
9	Benchimol et al. (2017)	2017	1999	North America	26530074	8278	7,07	3,77	10,37	1,58
10	Bentsen et al. (2002)	2002	1990	Europe	166456	3062	2,87	-1,78	7,52	1,46
11	Bitton et al. (2014)	2014	2001	North America	240937	1967	11,4	-5,08	27,88	5,18
12	Calkins et al. (1984)	1984	1973	North America	2174000	345	2,21	1,09	3,32	0,52
13	Cosgrove et al. (1996)	1996	1983	Europe	90000	60	3,25	1,32	5,18	0,61
14	El Mouzan et al. (2014)	2014	2003	Asia	7128085	323	0,27	0,18	0,35	0,04
15	El-Matary et al. (2014)	2014	1978	North America	283167	392	2,78	1,48	4,07	0,5
16	Fellows et al. (1990)	1990	1976	Europe	215300	268	3,15	-3,09	9,39	1,96
17	Ghione et al. (2018)	2018	1988	Europe	1312141	1225	3,08	0,91	5,25	0,94
18	Gottrand et al. (1991)	1991	1988	Europe	1054660	1350	2,1	.	.	.
19	Grieci, Büttner et al. (2009)	2009	1997	North America	352395	298	4,8	1,68	7,93	0,73
20	Haug et al. (2009)	2009	1992	Europe	403500	74	5,58	4,31	6,84	0,4
21	Henderson et al. (2012)	2012	1990	Europe	499142	645	3,83	1,78	5,88	0,93
22	Hildebrand et al. (1994)	1994	1983	Europe	288941	135	2,44	0,59	4,29	0,78
23	Hildebrand et al. (2003)	2003	1990	Europe	18000	191	5,54	3,36	7,72	0,96
24	Hong et al. (2018)	2018	2011	Asia	974638	124	1,71	0,93	2,49	0,3
25	Hope et al. (2012)	2012	2005	Europe	888310	349	2,3	.	.	.
26	Isa et. al. (2018)	2018	1987	Asia	343176	207	0,79	-0,89	2,46	0,39
27	Jacobsen B.A. et al. (2006)	2006	1978	Europe	486274	1217	1,45	-0,46	3,36	0,15
28	Jacobsen, C. et al. (2008)	2008	2002	Europe	430671	503	2,68	2,14	3,21	0,22
29	Jacobsen, C. et al. (2009)	2009	1996	Europe	117216	243	3,1	.	.	.
30	Jacobsen, C. et al. (2011)	2011	2007	Europe	668056	50	3,2	.	.	.
31	Jussila et al. (2012)	2012	2000	Europe	5180000	130	0,2	.	.	.
32	Karolewska-Bochenek et al. (2009)	2009	2002	Europe	9000000	491	0,82	-0,36	2	0,42
33	Kern et al. (2021)	2021	2000	Europe	436305	344	4,49	3,52	5,45	0,45
34	Kugathasan et al. (2003)	2003	2000	North America	540000	491	4,56	.	.	.
35	Kuo et al. (2015)	2015	2000	Asia	15818502	177	0,14	0,05	0,22	0,03
36	Kwak et al. (2019)	2019	2009	Asia	12127750	170	0,53	0,24	0,81	0,13
37	Larsen et al. (2016)	2016	1995	Europe	1000000	1820	6,53	0,66	12,39	1,84

38	Lehtinen et al. (2011)	2011	1993	Europe	1100000	1880	8,4	.	.	.
39	Lindberg et al. (1991)	1991	1983	Europe	164972	972	2,37	-6,35	11,09	2,03
40	Lindberg et al. (2000)	2000	1989	Europe	942898	246	1,3	.	.	.
41	Lindquist et al. (1984	1984	1971	Europe	56000	639	6,1	.	.	.
42	Lopez et al. (2018a)	2018	2015	Australia/ Pacif	110716	161	6,36	4,69	8,03	0,8
43	Lopez et al. (2018)	2018	2001	Australia/ Pacif	977800	51	3,5	.	.	.
44	Lovasz et al. (2014)	2014	1994	Europe	11400	863	3,6	-42,14	49,34	3,6
45	Malaty et al. (2010)	2010	1991	North America	1227491	358	1	-3,26	5,25	0,34
46	Malmborg et al. (2013)	2013	2002	Europe	188437	153	9,19	7,96	10,41	0,5
47	Martin-de -Carpi et al. (2013)	2013	1996	Europe	8160000	1712	1,12	-6,32	8,55	0,58
48	Muller et al. (2013)	2013	2007	Europe	1800000	2107	4,72	.	.	.
49	Nyhlin et al. (1986)	1986	1974	Europe	43861	271	3,63	-0,79	8,04	1,39
50	Olafsdottir et al. (1989)	1989	1984	Europe	198570	221	2,5	.	.	.
51	Ong et al. (2018)	2018	2004	Asia	910761	204	0,74	0,04	1,44	0,29
52	Orel et al. (2009)	2009	1994	Europe	283361	167	2,43	1,32	3,54	0,26
53	Phavichitr et al. (2003)	2003	1985	Australia/ Pacif	1080767	280	11,09	-15,07	37,24	6,08
54	Schwarz et al. (2017)	2017	2008	Europe	82588	261	5,4	-4,76	15,56	0,8
55	Shivashankar et al. (2017)	2017	1985	North America	33501	893	8,39	7,46	9,33	0,44
56	Sjoberg et al. (2014)	2014	2005	Europe	642117	279	6,9	-32,49	46,29	3,1
57	Stordal et al. (2004)	2004	1990	Europe	174482	471	2,7	.	.	.
58	Stowe et al. (1990)	1990	1980	North America	460497	1247	3,98	-0,55	8,51	1,42
59	Tourtelier et al. (2000)	2000	1994	Europe	618049	1377	1,6	1,17	2,03	0,15
60	Turunen et al. (2006)	2006	1995	Europe	75706	645	1,89	1,42	2,35	0,22
61	Urlep et al. (2014)	2014	2002	Europe	148702	145	4,69	3,92	5,47	0,36
62	Urlep et al. (2015)	2015	2002	Europe	414323	260	4,56	3,88	5,23	0,29
63	Urne et al. (2002)	2002	1998	Europe	421898	158	2,27	1,89	2,65	0,09
64	van der Zaag_Loonen et al. (2004)	2004	1999	Europe	3873000	98	2,1	.	.	.
65	Vicentin et al. (2017)	2017	2012	South America	11691156	107	0,17	.	.	.
66	Virta et al. (2017)	2017	2001	Europe	1235000	5379	7,96	6,44	9,48	0,75
67	Wang et al. (2013)	2013	2005	Asia	112013	482	2,96	1,75	4,17	0,44
68	Watson et al. (2002)	2002	1990	Europe	987470	130	3,3	-10,68	17,28	1,1
69	Wittig et al. (2019)	2019	2009	Europe	1074030	712	11,32	-1,86	24,5	4,75
70	Yamamoto-Furusho et al. (2019)	2019	2007	South America	40951452	1582	0,3	0,1	0,5	0,1
71	Yap et al. (2008)	2008	2002	Australia/ Pacif	885000	2073	1,9	.	.	.
72	Zyyani et al. 2017)	2017	2000	Asia	283951	370	4,37	-2,98	11,71	1,71
