# Supplementary material for: Correlation of Socioeconomic and Environmental Factors With Incidence of Crohn Disease in Children and Adolescents: Systematic Review and Meta-Regression
Source: JMIR Public Health Surveill. 2024 Mar 25;10:e48682. doi: 10.2196/48682 (PMC11002755; doi:10.2196/48682)
Supplement: Multimedia Appendix 4 [file publichealth_v10i1e48682_app4.pdf]

Supplement References of the found studies

Abramson, O., M. Durant, W. Mow, A. Finley, P. Kodali, A. Wong, V. Tavares, u. a. „Incidence, Prevalence, and Time Trends of Pediatric Inflammatory Bowel Disease in Northern California, 1996 to 2006". *J Pediatr* 157, Nr. 2 (August 2010): 233-239.e1. https://doi.org/10.1016/j.jpeds.2010.02.024.

Adamiak, T., D. Walkiewicz-Jedrzejczak, D. Fish, C. Brown, J. Tung, K. Khan, W. Faubion Jr., u. a. „Incidence, Clinical Characteristics, and Natural History of Pediatric IBD in Wisconsin: A Population-Based Epidemiological Study". *Inflamm Bowel Dis* 19, Nr. 6 (Mai 2013): 1218–23. https://doi.org/10.1097/MIB.0b013e318280b13e.

Ahmed, M., I. H. Davies, K. Hood, und H. R. Jenkins. „Incidence of Paediatric Inflammatory Bowel Disease in South Wales". *Arch Dis Child* 91, Nr. 4 (April 2006): 344–45. https://doi.org/10.1136/adc.2004.067413.

Armitage, E., H. E. Drummond, D. C. Wilson, und S. Ghosh. „Increasing Incidence of Both Juvenile-Onset Crohn's Disease and Ulcerative Colitis in Scotland". *Eur J Gastroenterol Hepatol* 13, Nr. 12 (Dezember 2001): 1439–47.

Ashton, J. J., A. E. Wiskin, S. Ennis, A. Batra, N. A. Afzal, und R. M. Beattie. „Rising Incidence of Paediatric Inflammatory Bowel Disease (PIBD) in Wessex, Southern England". *Arch Dis Child* 99, Nr. 7 (Juli 2014): 659–64. https://doi.org/10.1136/archdischild-2013-305419.

Auvin, Stéphane, Florence Molinié, Corinne Gower-Rousseau, Franck Brazier, Véronique Merle, Bruno Grandbastien, Raymond Marti, u. a. „Incidence, Clinical Presentation and Location at Diagnosis of Pediatric Inflammatory Bowel Disease: A Prospective Population-Based Study in Northern {France} (1988-1999)." *Journal of Pediatric Gastroenterology and Nutrition* 41, Nr. 1 (Juli 2005): 49–55. https://doi.org/10.1097/01.mpg.0000162479.74277.86.

Barton, J. R., S. Gillon, und A. Ferguson. „Incidence of Inflammatory Bowel Disease in Scottish Children between 1968 and 1983; Marginal Fall in Ulcerative Colitis, Three-Fold Rise in Crohn's Disease". *Gut* 30, Nr. 5 (Mai 1989): 618–22. https://doi.org/10.1136/gut.30.5.618.

Benchimol, E. I., C. N. Bernstein, A. Bitton, M. W. Carroll, H. Singh, A. R. Otley, M. Vutcovici, u. a. „Trends in Epidemiology of Pediatric Inflammatory Bowel Disease in Canada: Distributed Network Analysis of Multiple Population-Based Provincial Health Administrative Databases". *Am J Gastroenterol* 112, Nr. 7 (Juli 2017): 1120–34. https://doi.org/10.1038/ajg.2017.97.

Benchimol, E. I., D. R. Mack, G. C. Nguyen, S. B. Snapper, W. Li, N. Mojaverian, P. Quach, und A. M. Muise. „Incidence, Outcomes, and Health Services Burden of Very Early Onset Inflammatory Bowel Disease". *Gastroenterology* 147, Nr. 4 (Oktober 2014): 803-813.e7; quiz e14-5. https://doi.org/10.1053/j.gastro.2014.06.023.

Bentsen, B. S., B. Moum, und A. Ekbom. „Incidence of Inflammatory Bowel Disease in Children in Southeastern Norway: A Prospective Population-Based Study 1990-94". *Scand J Gastroenterol* 37, Nr. 5 (Mai 2002): 540–45. https://doi.org/10.1080/00365520252903080.

Bitton, A., M. Vutcovici, V. Patenaude, M. Sewitch, S. Suissa, und P. Brassard. „Epidemiology of Inflammatory Bowel Disease in Quebec: Recent Trends“. *Inflamm Bowel Dis* 20, Nr. 10 (Oktober 2014): 1770–76. https://doi.org/10.1097/mib.0000000000000162.
Calkins, B. M., A. M. Lilienfeld, C. F. Garland, und A. I. Mendeloff. „Trends in Incidence Rates of Ulcerative Colitis and Crohn’s Disease“. *Dig Dis Sci* 29, Nr. 10 (Oktober 1984): 913–20. https://doi.org/10.1007/bf01312480.

Cosgrove, M., R. F. Al-Atia, und H. R. Jenkins. „The Epidemiology of Paediatric Inflammatory Bowel Disease“. *Arch Dis Child* 74, Nr. 5 (Mai 1996): 460–61. https://doi.org/10.1136/adc.74.5.460.

El Mouzan, M. I., O. Saadah, K. Al-Saleem, M. Al Edreesi, M. Hasosah, A. Alanazi, M. Al Mofarreh, u. a. „Incidence of Pediatric Inflammatory Bowel Disease in Saudi Arabia: A Multicenter National Study“. *Inflamm Bowel Dis* 20, Nr. 6 (Juni 2014): 1085–90. https://doi.org/10.1097/mib.0000000000000048.

El-Matary, W., S. P. Moroz, und C. N. Bernstein. „Inflammatory Bowel Disease in Children of Manitoba: 30 Years’ Experience of a Tertiary Center“. *J Pediatr Gastroenterol Nutr* 59, Nr. 6 (Dezember 2014): 763–66. https://doi.org/10.1097/mpg.0000000000000525.

Fellows, I. W., J. G. Freeman, und G. K. Holmes. „Crohn’s Disease in the City of Derby, 1951-85“. *Gut* 31, Nr. 11 (November 1990): 1262–65. https://doi.org/10.1136/gut.31.11.1262.

Ghione, S., H. Sarter, M. Fumery, L. Armengol-Debeir, G. Savoye, D. Ley, C. Spyckerelle, u. a. „Dramatic Increase in Incidence of Ulcerative Colitis and Crohn’s Disease (1988-2011): A Population-Based Study of French Adolescents“. *Am J Gastroenterol* 113, Nr. 2 (Februar 2018): 265–72. https://doi.org/10.1038/ajg.2017.228.

Gottrand, F., J. F. Colombel, L. Moreno, J. L. Salomez, J. P. Farriaux, und A. Cortot. „[Incidence of inflammatory bowel diseases in children in the Nord-Pas-de-Calais region]“. *Arch Fr Pediatr* 48, Nr. 1 (Januar 1991): 25–28.

Grieci, T., und A. Butter. „The Incidence of Inflammatory Bowel Disease in the Pediatric Population of Southwestern Ontario“. *J Pediatr Surg* 44, Nr. 5 (Mai 2009): 977–80. https://doi.org/10.1016/j.jpedsurg.2009.01.038.

Haug, K., E. Schrumpf, J. F. Halvorsen, G. Fluge, E. Hamre, T. Hamre, und R. Skjollingstad. „Epidemiology of Crohn’s Disease in Western Norway. Study Group of Inflammatory Bowel Disease in Western Norway“. *Scand J Gastroenterol* 24, Nr. 10 (Dezember 1989): 1271–75. https://doi.org/10.3109/00365528909090798.

Henderson, P., R. Hansen, F. L. Cameron, K. Gerasimidis, P. Rogers, W. M. Bisset, E. L. Reynish, u. a. „Rising Incidence of Pediatric Inflammatory Bowel Disease in Scotland“. *Inflamm Bowel Dis* 18, Nr. 6 (Juni 2012): 999–1005. https://doi.org/10.1002/ibd.21797.

Hildebrand, H., M. Brydolf, L. Holmquist, I. Krantz, und B. Kristiansson. „Incidence and Prevalence of Inflammatory Bowel Disease in Children in South-Western Sweden“. *Acta Paediatr* 83, Nr. 6 (Juni 1994): 640–45. https://onlinelibrary.wiley.com/doi/abs/10.1111/j.16512227.1994.tb13098.x?sid=nlm%3Apubmed.

Hildebrand, H., Y. Finkel, L. Grahnquist, J. Lindholm, A. Ekbom, und J. Askling. „Changing Pattern of Paediatric Inflammatory Bowel Disease in Northern Stockholm 1990-2001“. *Gut* 52, Nr. 10 (Oktober 2003): 1432–34. https://doi.org/10.1136/gut.52.10.1432.

Hong, S. J., S. M. Cho, B. H. Choe, H. J. Jang, K. H. Choi, B. Kang, J. E. Kim, und J. H. Hwang. „Characteristics and Incidence Trends for Pediatric Inflammatory Bowel Disease in Daegu-Kyungpook Province in Korea: A Multi-Center Study“. *J Korean Med Sci* 33, Nr. 18 (30. April 2018): e132. https://doi.org/10.3346/jkms.2018.33.e132.

Hope, B., R. Shahdadpuri, C. Dunne, A. M. Broderick, T. Grant, M. Hamzawi, K. O’Driscoll, S. Quinn, S. Hussey, und B. Bourke. „Rapid Rise in Incidence of Irish Paediatric Inflammatory Bowel Disease“. *Arch Dis Child* 97, Nr. 7 (Juli 2012): 590–94. https://doi.org/10.1136/archdischild-2011-300651.

Isa, H. M., A. M. Mohamed, H. E. Al-Jowder, K. A. Matrook, und H. H. Althawadi. „Pediatric crohn’s disease in Bahrain“. *Oman Medical Journal* 33, Nr. 4 (2018): 299–308. https://doi.org/10.5001/omj.2018.56.

Jacobsen, B. A., J. Fallingborg, H. H. Rasmussen, K. R. Nielsen, A. M. Drewes, E. Puho, G. L. Nielsen, und H. T. Sorensen. „Increase in Incidence and Prevalence of Inflammatory Bowel Disease in Northern Denmark: A Population-Based Study, 1978-2002“. *Eur J Gastroenterol Hepatol* 18, Nr. 6 (Juni 2006): 601–6. https://doi.org/10.1097/00042737-200606000-00005.

Jakobsen, C., A. Paerregaard, P. Munkholm, J. Faerk, A. Lange, J. Andersen, M. Jakobsen, I. Kramer, J. Czernia-Mazurkiewicz, und V. Wewer. „Pediatric Inflammatory Bowel Disease: Increasing Incidence, Decreasing Surgery Rate, and Compromised Nutritional Status: A Prospective Population-Based Cohort Study 2007-2009“. *Inflamm Bowel Dis* 17, Nr. 12 (Dezember 2011): 2541–50. https://doi.org/10.1002/ibd.21654.

Jakobsen, C., A. Paerregaard, P. Munkholm, und V. Wewer. „Paediatric Inflammatory Bowel Disease during a 44-Year Period in Copenhagen County: Occurrence, Course and Prognosis--a Population-Based Study from the Danish Crohn Colitis Database“. *Eur J Gastroenterol Hepatol* 21, Nr. 11 (November 2009): 1291–1301. https://doi.org/10.1097/MEG.0b013e32832a4ed6.

Jakobsen, C., V. Wewer, F. Urne, J. Andersen, J. Faerk, I. Kramer, B. Stagegaard, B. Pilgaard, B. Weile, und A. Paerregaard. „Incidence of ulcerative colitis and Crohn’s disease in Danish children: Still rising or levelling out?“ *Journal of Crohn’s and Colitis* 2, Nr. 2 (2008): 152–57. https://doi.org/10.1016/j.crohns.2008.01.006.

Jussila, A., L. J. Virta, H. Kautiainen, M. Rekiaro, U. Nieminen, und M. A. Farkkila. „Increasing Incidence of Inflammatory Bowel Diseases between 2000 and 2007: A Nationwide Register Study in Finland“. *Inflamm Bowel Dis* 18, Nr. 3 (März 2012): 555–61. https://doi.org/10.1002/ibd.21695.

Karolewska-Bochenek, K., I. Lazowska-Przeorek, P. Albrecht, K. Grzybowska, J. Ryzko, K. Szamotulska, A. Radzikowski, u. a. „Epidemiology of inflammatory bowel disease among children in Poland“. *Digestion* 79, Nr. 2 (2009): 121–29. https://doi.org/10.1159/000209382.

Kern, Ivana, Olaf Schoffer, Wieland Kiess, Jobst Henker, Martin W. Laaß, Ulf Winkler, Jürgen Quietzsch, u. a. „Incidence Trends of Pediatric Onset Inflammatory Bowel Disease in the

Years 2000-2009 in Saxony, Germany-First Results of the Saxon Pediatric IBD Registry“. *PloS One* 16, Nr. 1 (2021): e0243774. https://doi.org/10.1371/journal.pone.0243774.

Kugathasan, S., R. H. Judd, R. G. Hoffmann, J. Heikenen, G. Telega, F. Khan, S. Weisdorf-Schindele, u. a. „Epidemiologic and Clinical Characteristics of Children with Newly Diagnosed Inflammatory Bowel Disease in Wisconsin: A Statewide Population-Based Study“. *J Pediatr* 143, Nr. 4 (Oktober 2003): 525–31. https://doi.org/10.1067/s0022-3476(03)00444-x.

Kuo, C. J., K. H. Yu, L. C. See, C. T. Chiu, M. Y. Su, C. M. Hsu, C. F. Kuo, M. J. Chiou, J. R. Liu, und H. W. Wang. „The Trend of Inflammatory Bowel Diseases in Taiwan: A Population-Based Study“. *Dig Dis Sci* 60, Nr. 8 (August 2015): 2454–62. https://doi.org/10.1007/s10620-015-3630-z.

Kwak, M. S., J. M. Cha, H. H. Lee, Y. S. Choi, S. I. Seo, K. J. Ko, D. I. Park, S. H. Kim, und T. J. Kim. „Emerging trends of inflammatory bowel disease in South Korea: A nationwide population-based study“. *Journal of Gastroenterology and Hepatology (Australia)* 34, Nr. 6 (2019): 1018–26. https://doi.org/10.1111/jgh.14542.

Ladas, S. D., E. Mallas, K. Giorgiotis, G. Karamanolis, D. Trigonis, A. Markadas, V. Sipsa, und S. A. Raptis. „Incidence of Ulcerative Colitis in Central Greece: A Prospective Study“. *World J Gastroenterol* 11, Nr. 12 (28. März 2005): 1785–87. https://doi.org/10.3748/wjg.v11.i12.1785.

Larsen, M. D., M. E. Baldal, R. G. Nielsen, J. Nielsen, K. Lund, und B. M. Norgard. „The Incidence of Crohn’s Disease and Ulcerative Colitis since 1995 in Danish Children and Adolescents <17 Years - Based on Nationwide Registry Data“. *Scand J Gastroenterol* 51, Nr. 9 (September 2016): 1100–1105. https://doi.org/10.3109/00365521.2016.1172340.

Lehtinen, Pieta, Merja Ashorn, Sari Iltanen, Raimo Jauhola, Pekka Jauhonen, Kaija-Leena Kolho, und Anssi Auvinen. „Incidence Trends of Pediatric Inflammatory Bowel Disease in Finland, 1987-2003, a Nationwide Study.“ *Inflammatory Bowel Diseases* 17, Nr. 8 (August 2011): 1778–83. https://doi.org/10.1002/ibd.21550.

Lindberg, E., und G. Jornerot. „The Incidence of Crohn’s Disease Is Not Decreasing in Sweden“. *Scand J Gastroenterol* 26, Nr. 5 (Mai 1991): 495–500. https://doi.org/10.3109/00365529108998572.

Lindberg, E., B. Lindquist, L. Holmquist, und H. Hildebrand. „Inflammatory Bowel Disease in Children and Adolescents in Sweden, 1984-1995“. *J Pediatr Gastroenterol Nutr* 30, Nr. 3 (März 2000): 259–64. https://doi.org/10.1097/00005176-200003000-00009.

Lindquist, B. L., G. Jarnerot, und G. Wickbom. „Clinical and Epidemiological Aspects of Crohn’s Disease in Children and Adolescents“. *Scand J Gastroenterol* 19, Nr. 4 (Juni 1984): 502–6.

Lopez, R. N., L. Appleton, R. B. Gearry, und A. S. Day. „Rising Incidence of Paediatric Inflammatory Bowel Disease in Canterbury, New Zealand, 1996-2015“. *Journal of Pediatric Gastroenterology and Nutrition* 66, Nr. 2 (2018): e45–50. https://doi.org/10.1097/MPG.0000000000001688.

Lopez, R. N., H. M. Evans, L. Appleton, J. Bishop, S. Chin, S. Mouat, R. B. Gearry, und A. S. Day. „Prospective Incidence of Paediatric Inflammatory Bowel Disease in New Zealand in 2015: Results from the Paediatric Inflammatory Bowel Disease in New Zealand (PINZ) Study". *Journal of Pediatric Gastroenterology and Nutrition* 66, Nr. 5 (2018): e122–26. https://doi.org/10.1097/MPG.0000000000001806.

Lovasz, B. D., L. Lakatos, A. Horvath, T. Pandur, Z. Erdelyi, M. Balogh, I. Szipocs, u. a. „Incidence Rates and Disease Course of Paediatric Inflammatory Bowel Diseases in Western Hungary between 1977 and 2011". *Dig Liver Dis* 46, Nr. 5 (Mai 2014): 405–11. https://doi.org/10.1016/j.dld.2013.12.013.

Malaty, H. M., X. Fan, A. R. Opekun, C. Thibodeaux, und G. D. Ferry. „Rising Incidence of Inflammatory Bowel Disease among Children: A 12-Year Study". *J Pediatr Gastroenterol Nutr* 50, Nr. 1 (Januar 2010): 27–31. https://doi.org/10.1097/MPG.0b013e3181b99baa.

Malmborg, P., L. Grahnquist, J. Lindholm, S. Montgomery, und H. Hildebrand. „Increasing Incidence of Paediatric Inflammatory Bowel Disease in Northern Stockholm County, 2002-2007". *J Pediatr Gastroenterol Nutr* 57, Nr. 1 (Juli 2013): 29–34. https://doi.org/10.1097/MPG.0b013e31828f21b4.

Martin-de-Carpi, J., E. Ramos, S. Jimenez, M. J. Martinez-Gomez, E. Medina, J. Serrano, E. Ricart, u. a. „Increasing incidence of pediatric inflammatory bowel disease in Spain (1996-2009): The SPIRIT registry". *Inflammatory Bowel Diseases* 19, Nr. 1 (2013): 73–80. https://doi.org/10.1002/ibd.22980.

Muller, K. E., P. L. Lakatos, A. Arato, J. B. Kovacs, A. Varkonyi, D. Szucs, E. Szakos, u. a. „Incidence, Paris Classification, and Follow-up in a Nationwide Incident Cohort of Pediatric Patients with Inflammatory Bowel Disease". *J Pediatr Gastroenterol Nutr* 57, Nr. 5 (November 2013): 576–82. https://doi.org/10.1097/MPG.0b013e31829f7d8c.

Nyhlin, H., und A. Danielsson. „Incidence of Crohn's Disease in a Defined Population in Northern Sweden, 1974-1981". *Scand J Gastroenterol* 21, Nr. 10 (Dezember 1986): 1185–92. https://doi.org/10.3109/00365528608996441.

Olafsdottir, E. J., G. Fluge, und K. Haug. „Chronic Inflammatory Bowel Disease in Children in Western Norway". *J Pediatr Gastroenterol Nutr* 8, Nr. 4 (Mai 1989): 454–58. https://doi.org/10.1097/00005176-198905000-00006.

Ong, C., M. M. Aw, M. J. Liwanag, S. H. Quak, und K. B. Phua. „Rapid Rise in the Incidence and Clinical Characteristics of Pediatric Inflammatory Bowel Disease in a South-East Asian Cohort in Singapore, 1994-2015". *J Dig Dis* 19, Nr. 7 (Juli 2018): 395–403. https://doi.org/10.1111/1751-2980.12641.

Orel, R., T. Kamhi, G. Vidmar, und P. Mamula. „Epidemiology of Pediatric Chronic Inflammatory Bowel Disease in Central and Western Slovenia, 1994-2005". *J Pediatr Gastroenterol Nutr* 48, Nr. 5 (Mai 2009): 579–86. https://doi.org/10.1097/MPG.0b013e318164d903.

Phavichitr, N., D. J. Cameron, und A. G. Catto-Smith. „Increasing Incidence of Crohn's Disease in Victorian Children". *J Gastroenterol Hepatol* 18, Nr. 3 (März 2003): 329–32. https://onlinelibrary.wiley.com/doi/pdf/10.1046/j.1440-1746.2003.02975.x.

Schwarz, J., J. Sykora, D. Cvalinova, R. Pomahacova, J. Kleckova, M. Kryl, und P. Vcelak. „Inflammatory Bowel Disease Incidence in Czech Children: A Regional Prospective Study, 2000-2015“. *World J Gastroenterol* 23, Nr. 22 (14. Juni 2017): 4090–4101. https://doi.org/10.3748/wjg.v23.i22.4090.

Shivashankar, Raina, William J. Tremaine, W. Scott Harmsen, und Edward V. Jr Loftus. „Incidence and Prevalence of Crohn’s Disease and Ulcerative Colitis in Olmsted County, Minnesota From 1970 Through 2010.“ *Clinical Gastroenterology and Hepatology : The Official Clinical Practice Journal of the American Gastroenterological Association* 15, Nr. 6 (Juni 2017): 857–63. https://doi.org/10.1016/j.cgh.2016.10.039.

Sjoberg, D., T. Holmstrom, M. Larsson, A. L. Nielsen, L. Holmquist, A. Ekbom, und A. Ronnblom. „Incidence and Clinical Course of Crohn’s Disease during the First Year - Results from the IBD Cohort of the Uppsala Region (ICURE) of Sweden 2005-2009“. *J Crohns Colitis* 8, Nr. 3 (März 2014): 215–22. https://doi.org/10.1016/j.crohns.2013.08.009.

Stordal, K., J. Jahnsen, B. S. Bentsen, und B. Moum. „Pediatric Inflammatory Bowel Disease in Southeastern Norway: A Five-Year Follow-up Study“. *Digestion* 70, Nr. 4 (2004): 226–30. https://doi.org/10.1159/000082893.

Stowe, S. P., S. R. Redmond, J. M. Stormont, A. N. Shah, L. N. Chessin, H. L. Segal, und W. Y. Chey. „An Epidemiologic Study of Inflammatory Bowel Disease in Rochester, New York. Hospital Incidence“. *Gastroenterology* 98, Nr. 1 (Januar 1990): 104–10. https://doi.org/10.1016/0016-5085(90)91297-j.

Tourtelier, Y., A. Dabadie, I. Tron, J. L. Alexandre, M. Robaskiewicz, E. Cruchant, J. A. Seyrig, D. Heresbach, und J. F. Bretagne. „[Incidence of inflammatory bowel disease in children in Brittany (1994-1997). Breton association of study and research on digestive system diseases (Abermad)]“. *Arch Pediatr* 7, Nr. 4 (April 2000): 377–84. https://doi.org/10.1016/s0929-693x(00)88832-6.

Turunen, P., K. L. Kolho, A. Auvinen, S. Iltanen, H. Huhtala, und M. Ashorn. „Incidence of Inflammatory Bowel Disease in Finnish Children, 1987-2003“. *Inflamm Bowel Dis* 12, Nr. 8 (August 2006): 677–83. https://doi.org/10.1097/00054725-200608000-00002.

Urlep, D., R. Blagus, und R. Orel. „Incidence Trends and Geographical Variability of Pediatric Inflammatory Bowel Disease in Slovenia: A Nationwide Study“. *Biomed Res Int* 2015 (2015): 921730. https://doi.org/10.1155/2015/921730.

Urlep, D., T. K. Trop, R. Blagus, und R. Orel. „Incidence and Phenotypic Characteristics of Pediatric IBD in Northeastern Slovenia, 2002-2010“. *J Pediatr Gastroenterol Nutr* 58, Nr. 3 (März 2014): 325–32. https://doi.org/10.1097/mpg.0000000000000207.

Urne, F. U., und A. Paerregaard. „[Chronic inflammatory bowel disease in children. An epidemiological study from eastern Denmark 1998-2000]“. *Ugeskr Laeger* 164, Nr. 49 (2. Dezember 2002): 5810–14.

Vicentin, R., M. Wagener, A. B. Pais, M. Contreras, und M. Orsi. „One-Year Prospective Registry of Inflammatory Bowel Disease in the Argentine Pediatric Population“. *Arch Argent Pediatr* 115, Nr. 6 (1. Dezember 2017): 533–40. https://doi.org/10.5546/aap.2017.eng.533.

Virta, L. J., M. M. Saarinen, und K. L. Kolho. „Inflammatory Bowel Disease Incidence Is on the Continuous Rise Among All Paediatric Patients Except for the Very Young: A Nationwide Registry-Based Study on 28-Year Follow-Up“. *J Crohns Colitis* 11, Nr. 2 (Februar 2017): 150–56. https://doi.org/10.1093/ecco-jcc/jjw148.

Wang, X. Q., Y. Zhang, C. D. Xu, L. R. Jiang, Y. Huang, H. M. Du, und X. J. Wang. „Inflammatory Bowel Disease in Chinese Children: A Multicenter Analysis over a Decade from Shanghai“. *Inflamm Bowel Dis* 19, Nr. 2 (Februar 2013): 423–28. https://doi.org/10.1097/MIB.0b013e318286f9f2.

Watson, A. J., A. T. Johnston, P. M. Barker, G. G. Youngson, W. M. Bisset, und A. A. Mahomed. „The Presentation and Management of Juvenile-Onset Chronic Inflammatory Bowel Disease in Northeastern Scotland“. *J Pediatr Surg* 37, Nr. 1 (Januar 2002): 83–86. https://doi.org/10.1053/jpsu.2002.29434.

Yamamoto-Furusho, J. K., A. Sarmiento-Aguilar, J. J. Toledo-Maurino, K. E. Bozada-Gutierrez, F. J. Bosques-Padilla, M. A. Martinez-Vazquez, V. Marroquin-Jimenez, u. a. „Incidence and Prevalence of Inflammatory Bowel Disease in Mexico from a Nationwide Cohort Study in a Period of 15 Years (2000-2017)“. *Medicine (Baltimore)* 98, Nr. 27 (Juli 2019): e16291. https://doi.org/10.1097/md.0000000000016291.

Yap, J., A. Wesley, S. Mouat, und S. Chin. „Paediatric Inflammatory Bowel Disease in New Zealand“. *N Z Med J* 121, Nr. 1283 (3. Oktober 2008): 19–34.

Zaag-Loonen, H. J. van der, M. Casparie, J. A. Taminiau, J. C. Escher, R. R. Pereira, und H. H. Derkx. „The Incidence of Pediatric Inflammatory Bowel Disease in the Netherlands: 1999-2001“. *J Pediatr Gastroenterol Nutr* 38, Nr. 3 (März 2004): 302–7. https://doi.org/10.1097/00005176-200403000-00014.

Zayyani, N. R., H. M. Malaty, und D. Y. Graham. „Increasing Incidence of Crohn’s Disease with Familial Clustering in the Kingdom of Bahrain: A 25-Year Population-Based Study“. *Inflamm Bowel Dis* 23, Nr. 2 (Februar 2017): 304–9. https://doi.org/10.1097/mib.0000000000001016.
