# Supplementary figures and images for: Correlation of Socioeconomic and Environmental Factors With Incidence of Crohn Disease in Children and Adolescents: Systematic Review and Meta-Regression
Source: JMIR Public Health Surveill. 2024 Mar 25;10:e48682. doi: 10.2196/48682 (PMC11002755; doi:10.2196/48682)

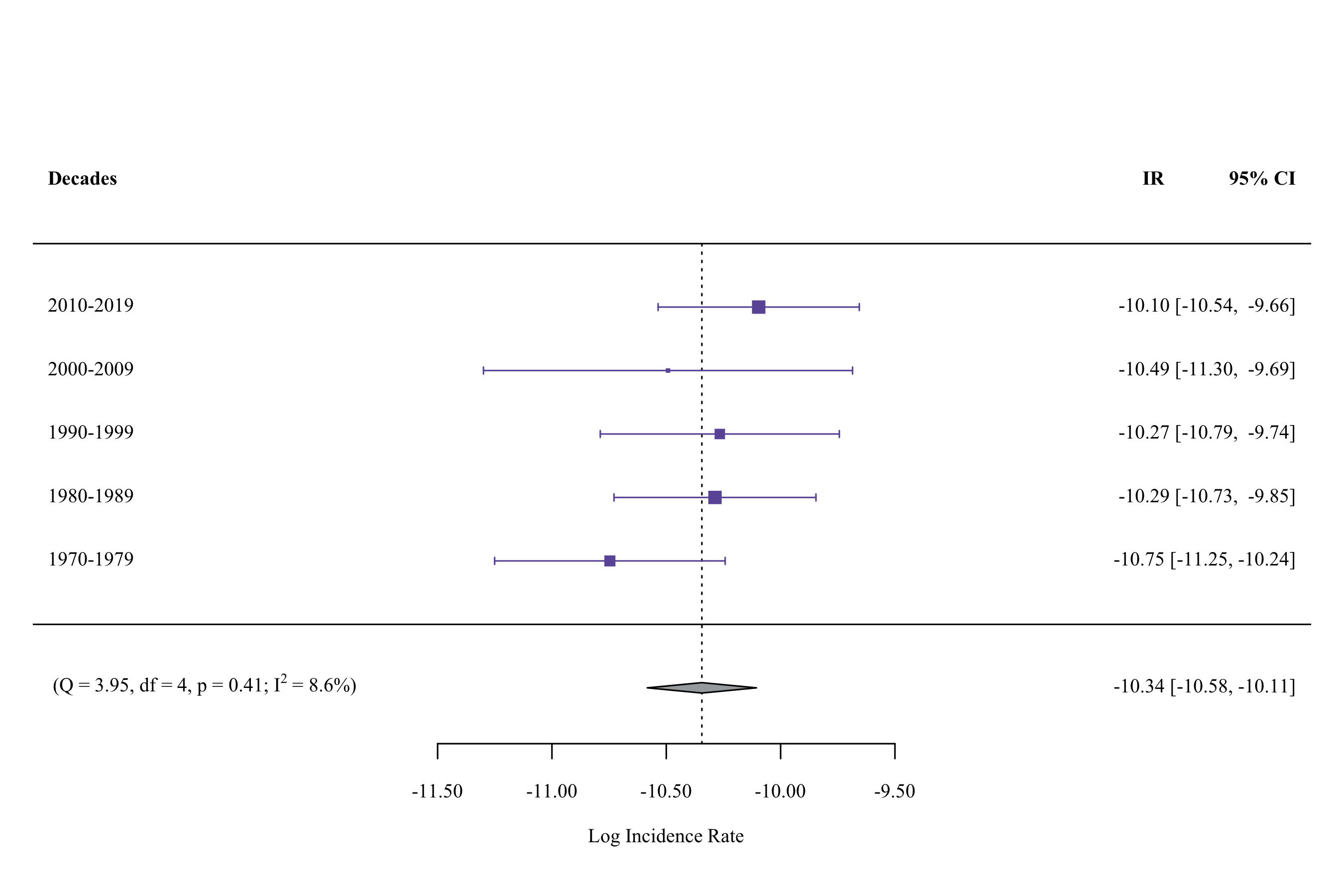

Supplement: Multimedia Appendix 6 [file publichealth_v10i1e48682_app6.png]

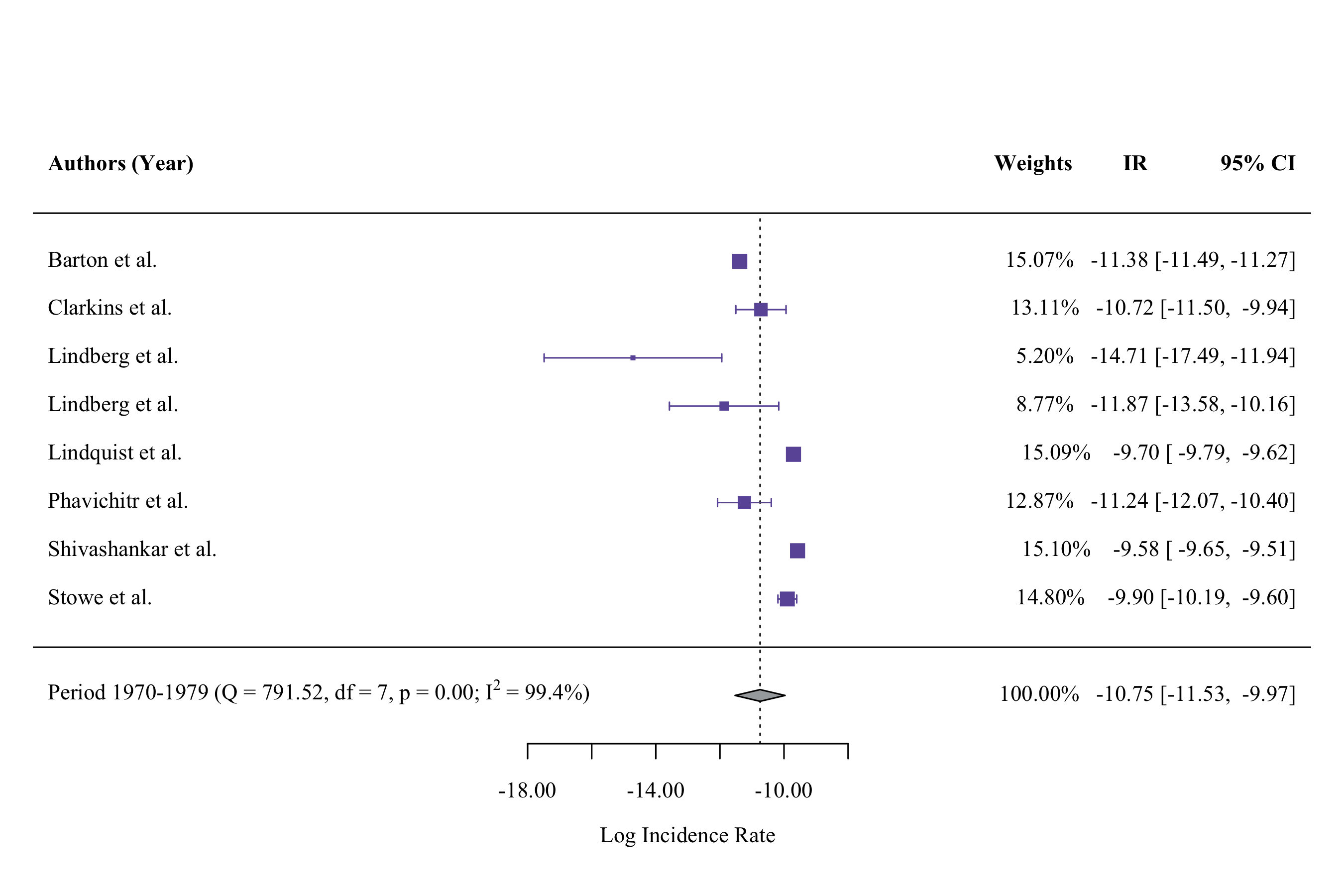

Supplement: Multimedia Appendix 7 [file publichealth_v10i1e48682_app7.png]

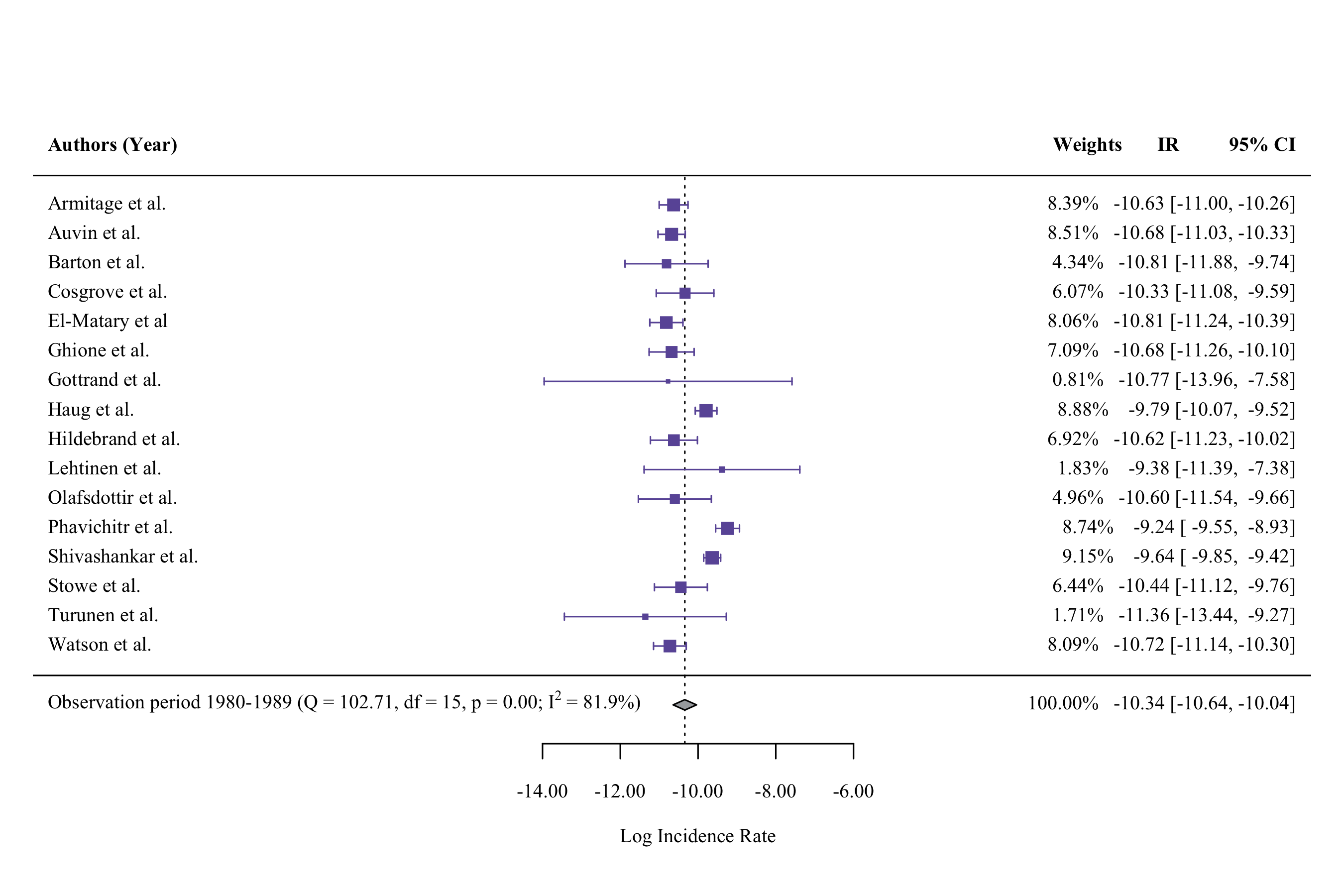

Supplement: Multimedia Appendix 8 [file publichealth_v10i1e48682_app8.png]

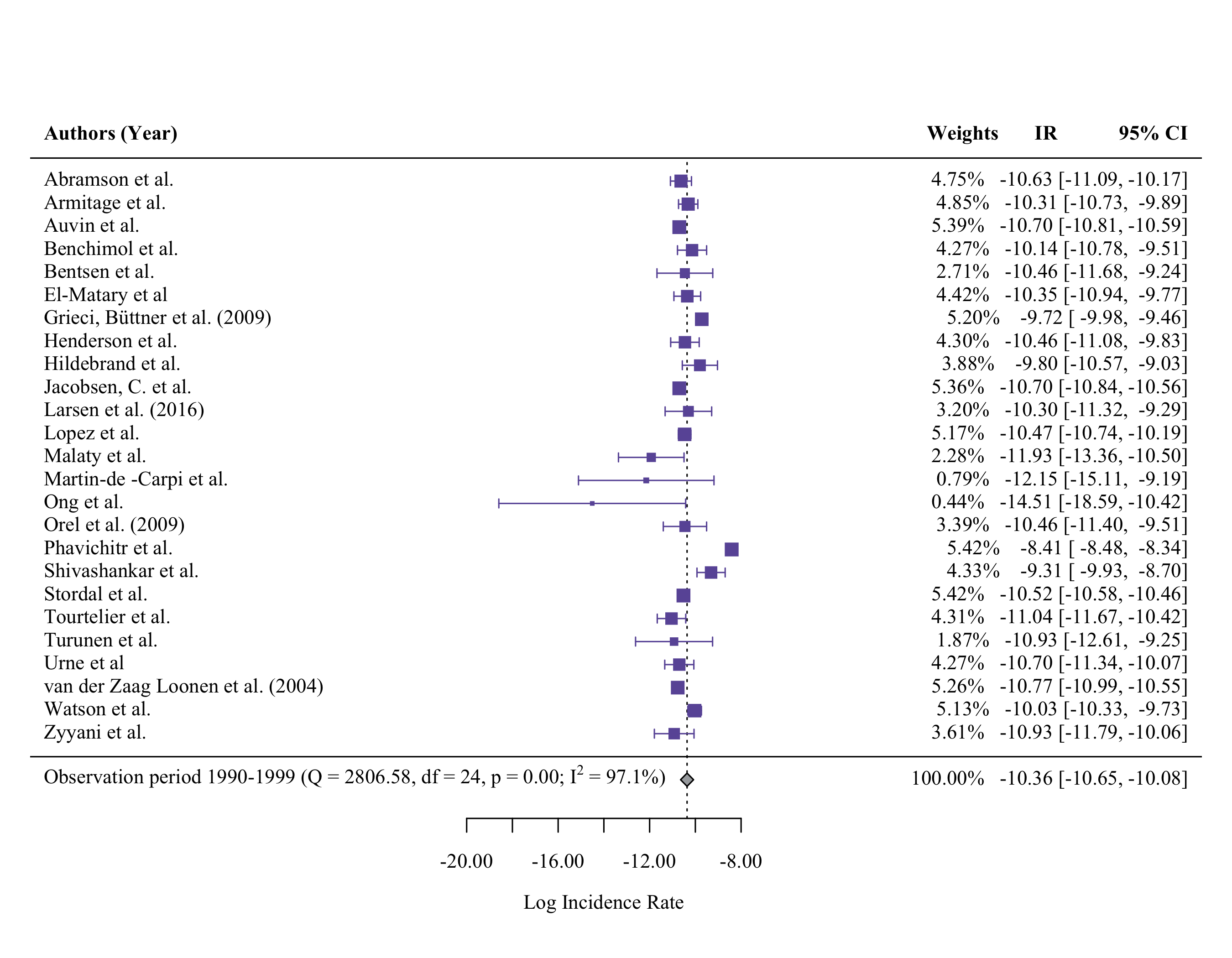

Supplement: Multimedia Appendix 9 [file publichealth_v10i1e48682_app9.png]

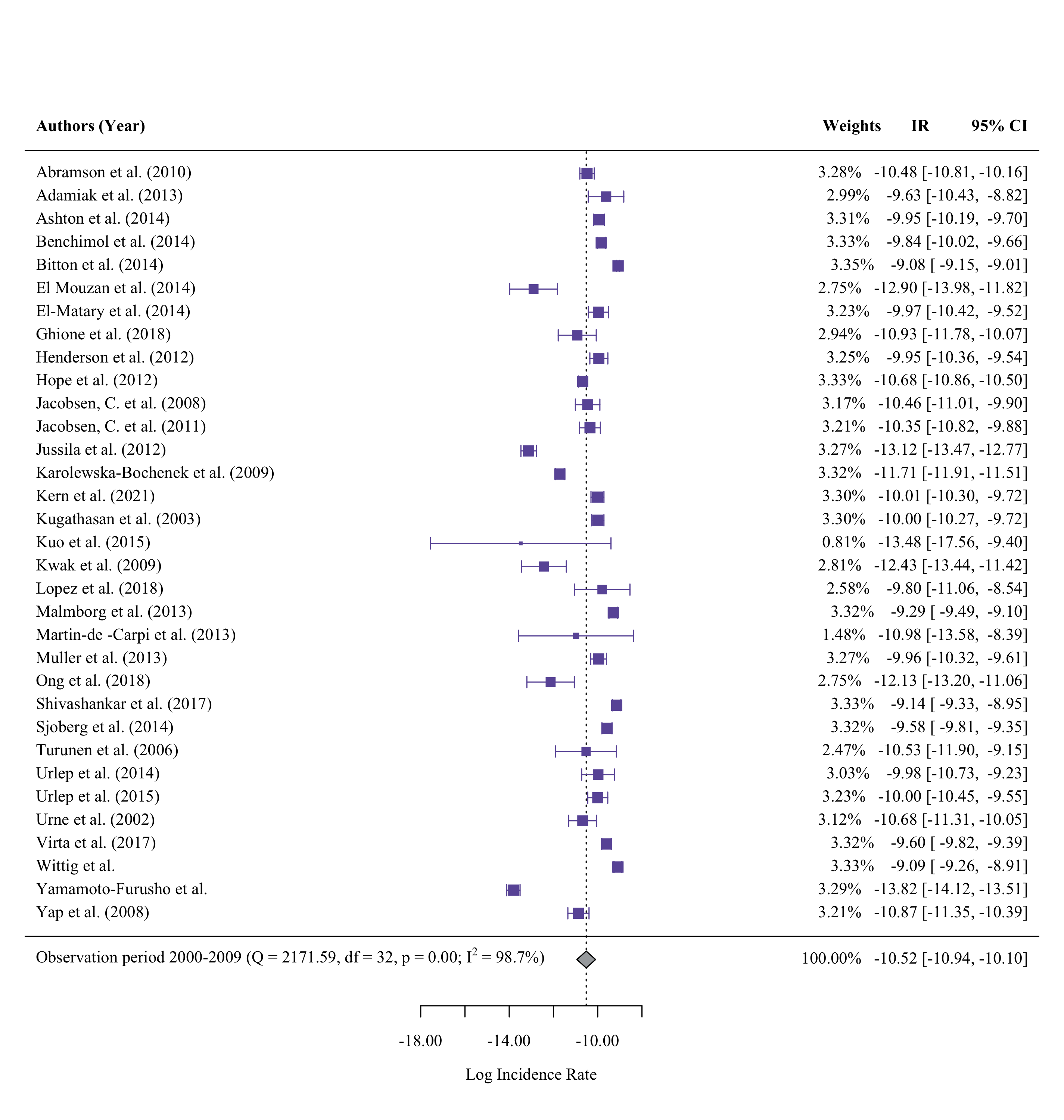

Supplement: Multimedia Appendix 10 [file publichealth_v10i1e48682_app10.png]

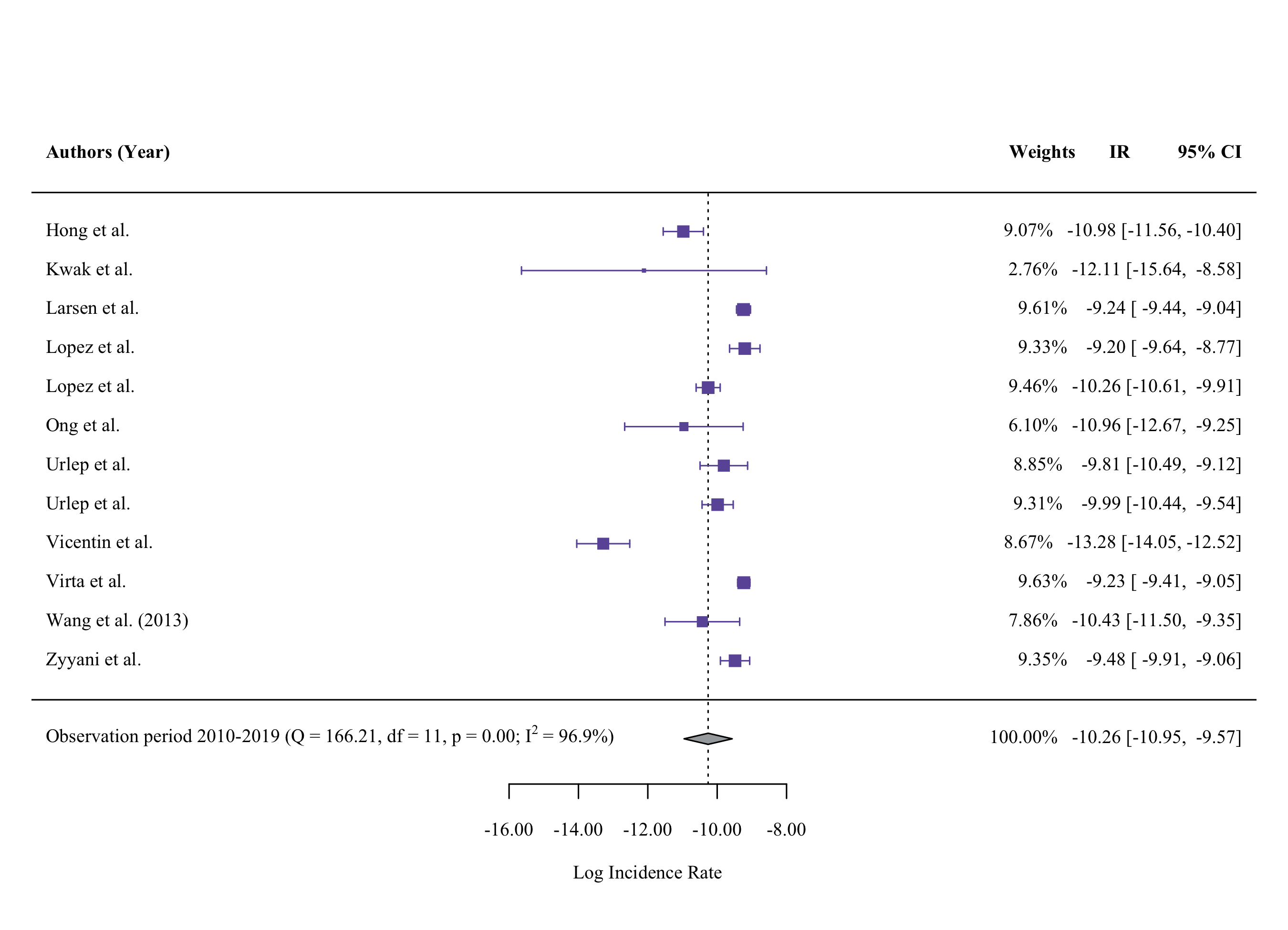

Supplement: Multimedia Appendix 11 [file publichealth_v10i1e48682_app11.png]

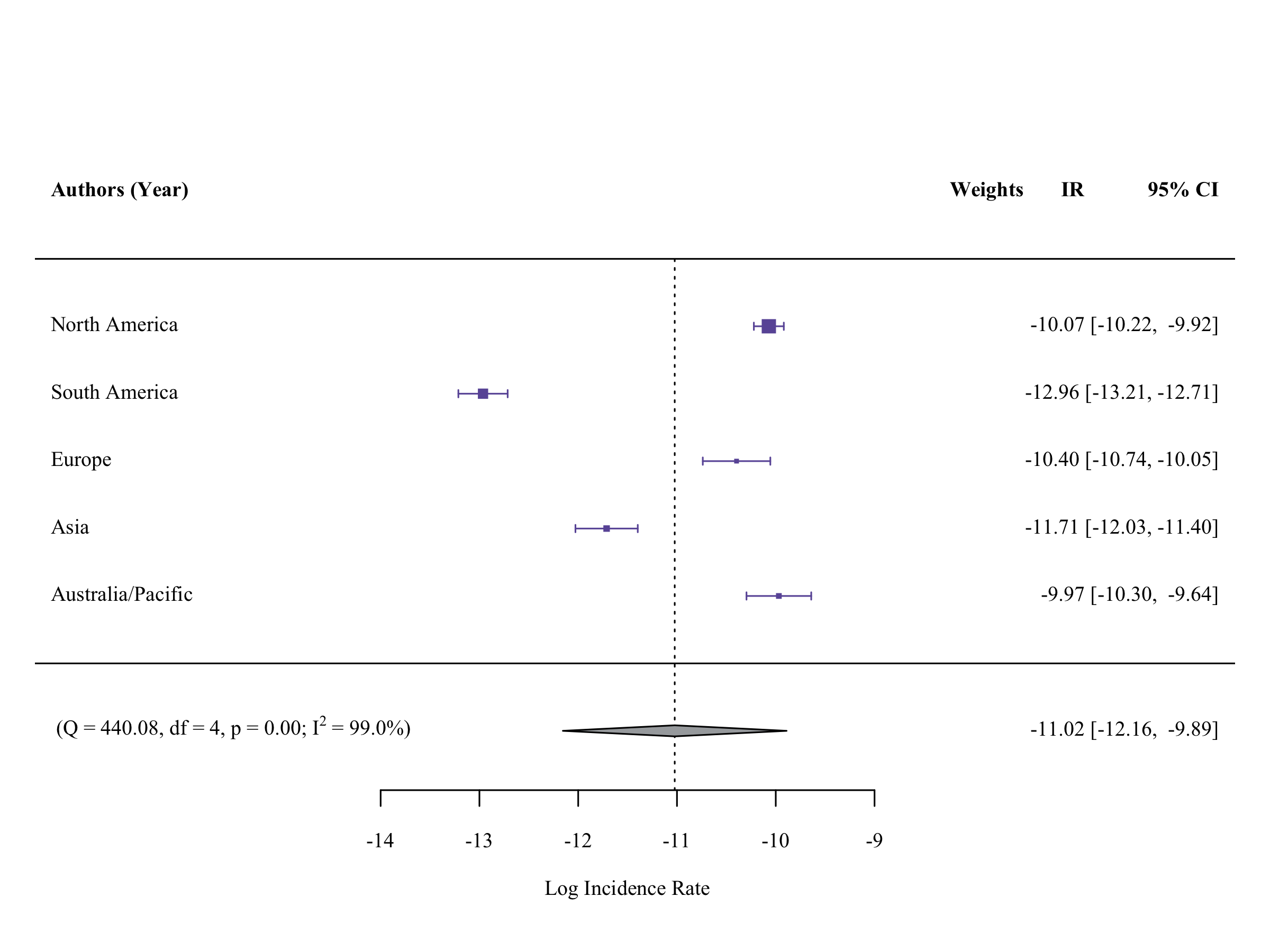

Supplement: Multimedia Appendix 12 [file publichealth_v10i1e48682_app12.png]

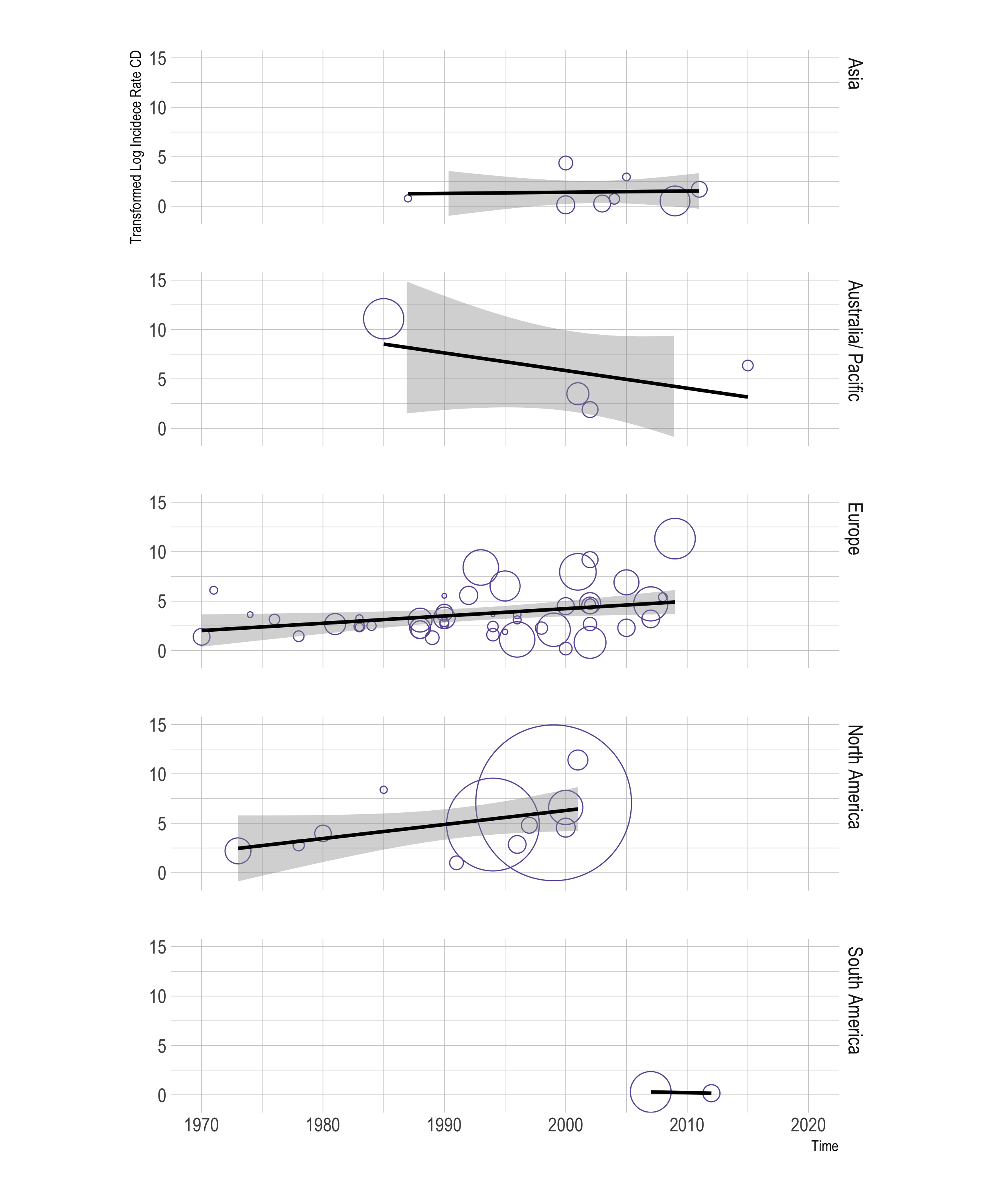

Supplement: Multimedia Appendix 13 [file publichealth_v10i1e48682_app13.png]
