# Supplementary material for: Correlation of Socioeconomic and Environmental Factors With Incidence of Crohn Disease in Children and Adolescents: Systematic Review and Meta-Regression
Source: JMIR Public Health Surveill. 2024 Mar 25;10:e48682. doi: 10.2196/48682 (PMC11002755; doi:10.2196/48682)
Supplement: Multimedia Appendix 14 [file publichealth_v10i1e48682_app14.pdf]

Continent	Estimate	SE	z-val	*P*-val	95%-CI	I^2	R^2	Exp. Coef.[a]
				model: IR ~(Time)				
Asia	0.02	0.07	0.27	.79	-0.11 – 0.15	95.56 %	0.00 %	1.018
Australia/ Pacific	-0.03	0.04	-0.66	.51	-0.10 – 0.05	92.88 %	0.00%	0.973
Europe	0.02	0.01	1.45	.14	-0.01 – 0.04	92.04 %	4.19%	1.017
North America	0.03	0.02	2.01	.04*	0.00 – 0.07	96.29 %	29.93 %	1.035
South America[1]	-	-	-	-	-	-	-	-
